# Internalized Amyloid-β (1-42) Peptide Inhibits the Store-Operated Calcium Entry in HT-22 Cells

**DOI:** 10.3390/ijms232012678

**Published:** 2022-10-21

**Authors:** Joana Poejo, Yolanda Orantos-Aguilera, Francisco Javier Martin-Romero, Ana Maria Mata, Carlos Gutierrez-Merino

**Affiliations:** 1 Instituto de Biomarcadores de Patologías Moleculares (IBPM), Universidad de Extremadura, 06006 Badajoz, Spain; 2 Departamento de Bioquímica y Biología Molecular y Genética, Facultad de Ciencias, Universidad de Extremadura, 06006 Badajoz, Spain

**Keywords:** amyloid-β(1-42), store-operated calcium entry, STIM1, calmodulin, endoplasmic reticulum, HT-22 cells, Alzheimer’s disease

## Abstract

Dysregulation in calcium signaling pathways plays a major role in the initiation of Alzheimer’s disease (AD) pathogenesis. Accumulative experimental evidence obtained with cellular and animal models, as well as with AD brain samples, points out the high cytotoxicity of soluble small oligomeric forms of amyloid-β peptides (Aβ) in AD. In recent works, we have proposed that Aβ-calmodulin (CaM) complexation may play a major role in neuronal Ca^2+^ signaling, mediated by CaM-binding proteins (CaMBPs). STIM1, a recognized CaMBP, plays a key role in store-operated calcium entry (SOCE), and it has been shown that the SOCE function is diminished in AD, resulting in the instability of dendric spines and enhanced amyloidogenesis. In this work, we show that 2 and 5 h of incubation with 2 μM Aβ(1-42) oligomers of the immortalized mouse hippocampal cell line HT-22 leads to the internalization of 62 ± 11 nM and 135 ± 15 nM of Aβ(1-42), respectively. Internalized Aβ(1-42) oligomers colocalize with the endoplasmic reticulum (ER) and co-immunoprecipitated with STIM1, unveiling that this protein is a novel target of Aβ. Fluorescence resonance energy transfer measurements between STIM1 tagged with a green fluorescent protein (GFP) and Aβ(1-42)-HiLyte™-Fluor555 show that STIM1 can bind nanomolar concentrations of Aβ(1-42) oligomers at a site located close to the CaM-binding site in STIM1. Internalized Aβ(1-42) produced dysregulation of the SOCE in the HT-22 cells before a sustained alteration of cytosolic Ca^2+^ homeostasis can be detected, and is elicited by only 2 h of incubation with 2 μM Aβ(1-42) oligomers. We conclude that Aβ(1-42)-induced SOCE dysregulation in HT-22 cells is caused by the inhibitory modulation of STIM1, and the partial activation of ER Ca^2+^-leak channels.

## 1. Introduction

There is a growing body of evidence demonstrating that dysregulation in signaling pathways that handle Ca^2+^ plays a major role in the initiation of Alzheimer’s disease (AD) pathogenesis. According to the “calcium hypothesis of brain aging and AD”, proposed first by Khachaturian [1], sustained changes in Ca^2+^ homeostasis could be a common pathway for aging and the neuropathological changes associated with AD. In recent decades, several studies have confirmed that Ca^2+^ signaling is upregulated in AD [2]. For example, Kuchibhotla et al. [3] reported higher basal Ca^2+^ concentrations in neurons close to Amyloid-β (Aβ) in amyloid precursor protein transgenic mice, compared with the wild-type mice. Likewise, the resting levels of Ca^2+^ in cortical neurons in a triple-transgenic mouse model of AD (3xTg-AD) were twice that found in non-transgenic animals [4]. Moreover, presenilin 1 and 2 mutations, which contribute to 90% of early-onset familial AD (counting for less than 5% of all cases) are related to the dysregulation of cytoplasmic Ca^2+^ homeostasis in AD neurons. It has been shown that a Ca^2+^ imbalance due to presenilin mutations takes place before the formation of Aβ plaques or tau aggregation in AD, suggesting once more that the dysregulation of Ca^2+^ may be the proximal origin of the pathology [5]. Furthermore, our recent works [6,7,8] highlight a novel function of calmodulin (CaM), i.e., the buffering of free Aβ concentrations in the low nanomolar range in neurons, due to the high concentration of CaM in neurons and its high affinity for neurotoxic Aβ peptides (dissociation constant ≈ 1 nM) [9]. Therefore, the concentration of Aβ-CaM complexes within neurons will increase as a function of time after the induction of Aβ production, and free Aβ will rise sharply when accumulated Aβ exceeds all the available CaM. Thus, Aβ-CaM complexation could also play a major role in neuronal Ca^2+^ signaling mediated by CaM-binding proteins, which play major roles in neuronal activity and excitability, as reviewed in [7].

For many years, the neurodegeneration of AD was attributed to the abnormal accumulation of insoluble fibrils (amyloid cascade hypothesis of AD) due to the observation of Aβ plaques in various regions of the brain [10]. However, accumulative studies in cellular models [11,12], mouse AD models [13], and AD brain tissue [14,15] have proposed that the small and soluble oligomeric forms of Aβ (e.g., dimers, trimers, tetramers, dodecamers, and higher oligomers) are the main cytotoxic forms in AD, and Aβ plaques could serve as reservoirs for the assembly of neurotoxic Aβ oligomers [16]. In a study performed in rat brains in vivo, it was demonstrated that the rats infused with soluble Aβ(1-42) oligomers exhibited more neurodegeneration, a greater inflammatory response, and a much greater decline in spatial learning and memory than the rats that received insoluble Aβ(1-42) fibrils [17]. In another study, it has been demonstrated that Aβ oligomers injected in the lateral ventricle of rats and macaques diffused into the brain and accumulated in several regions associated with memory and cognitive functions, with the consequent induction of tau phosphorylation, microglial activation, and synaptic loss in macaques where Aβ oligomers accumulated without the detection of fibrillar Aβ aggregates [18]. The conclusions of this work were revealed to be of extreme importance to understanding the main mechanism implicated in AD pathogenesis since humans and macaque brains share significant similarities [18]. Additionally, the mechanistic studies performed in different neuronal cell lines have indicated that the exogenous addition of the oligomeric forms of Aβ(1-40)/(1-42) to the extracellular medium leads to the internalization of Aβ in neurons, showing an intraneuronal subcellar distribution between mitochondria, lysosomes/endosomes, and the ER [19,20,21,22,23].

The principal function of the store-operated calcium entry (SOCE) is to refill the intracellular Ca^2+^ stores to preserve the primary source of intracellular Ca^2+^ and to therefore maintain a favorable environment for protein folding in the endoplasmic reticulum (ER) lumen [24]. In recent years, accumulative evidence suggested that SOCE is also involved in the excitability of neurons by playing a major role in axonal growth and synaptic plasticity [25]. The SOCE mechanism dysregulation has been associated with the disruption of intracellular Ca^2+^ signaling in neurons and consequently plays an active role in the pathogenesis of different neurogenerative diseases like Parkinson’s disease, AD or ischemic stroke, and Huntington’s disease [26]. Accumulating evidence has shown that the SOCE function is diminished in AD, resulting in the instability of dendric spines and enhanced amyloidogenesis [27]. In addition, we have reported that the levels of stromal interaction molecule 1 (STIM1) expression in brain tissues of the medium frontal gyrus decreased with the progression of neurodegeneration [28]. Other studies have shown attenuated Ca^2+^ entry in skin fibroblasts from familial AD patients [29,30]. Furthermore, it has been demonstrated that STIM1 is cleaved by the presenilin 1-secretase, leading to the dysregulation of Ca^2+^ homeostasis in SH-SY5Y and fibroblasts from familial AD patients [31]. According to Pannaccione et al. [32], there is also an excess of Ca^2+^ release from intracellular stores such as the ER both in AD animal models and patients. It was observed in early and pre-symptomatic mouse models of AD stages that ryanodine receptors (RyR) mediated Ca^2+^ upregulation in synaptic compartments, which are associated with altered synaptic homeostasis and network depression [33,34]. The dysregulation of RyR Ca^2+^ signaling leads to synaptic dysfunction at both pre- and post-synaptic levels, affecting the neuronal excitability and the short- and long-term plasticity mechanism, which are involved in learning and memory [3,35,36,37]. Furthermore, Ferreiro et al. [38] demonstrated that Aβ(1-40) induced an early increase in intracellular free Ca^2+^ levels due to the release of Ca^2+^ from the ER through both the inositol-1,4,5-trisphosphate receptor (IP3R) and RyR, leading to the consequent perturbation of Ca^2+^ homeostasis. All these findings suggest that some of the SOCE components, namely STIMs, RyR, and IP3R, are involved in the early dysregulation of Ca^2+^ homeostasis in AD and highlight the importance of further mechanistic studies to dissect the molecular pathways for the development of new drugs against early stages of AD progression associated with SOCE Ca^2+^ dysregulation. The immortalized mouse hippocampal cell line HT-22 used in this work expresses the RyR2 isoform [39] and all the three isotypes of IP3R [40].

SOCE is based on the influx of Ca^2+^ from the extracellular environment through channels of the plasma membrane and the refiling of Ca^2+^ in the ER when their levels decrease because of a release into the cytoplasm [41,42]. The basic components of the SOCE are well known and include a mechanism for Ca^2+^ stores depletion through the ligand-gated Ca^2+^ channels RyR and IP3R; a Ca^2+^ sensor in the ER that also serves as an activator of the plasma membrane channel (STIM1 or 2); and the store-operated channel in the plasma membrane (ORAI1, 2 or 3) [43]. The depletion of the ER Ca^2+^ stores causes the oligomerization of STIMs and their movement toward the ER–plasma membrane junctions [44]. In these junctions, STIMs form complexes with proteins of Ca^2+^ release-activated channels that are formed by ORAIs or store-operated channels, which are composed of ORAIs and transient receptor potential cation channels [44]. The activation of these channels causes the influx of Ca^2+^ ions through the plasma membrane from the extracellular medium into the cytoplasm and then to the ER lumen through the Sarco/endoplasmic reticulum Ca^2+^-adenosine triphosphatase (SERCA) pump [44]. Whereas STIM1 is ubiquitously expressed in most tissues, including neurons [45], and is involved in the regulation of SOCE, STIM2 appears to be more concentrated in neuronal tissues and has a lower affinity for Ca^2+^ sensing compared with STIM1, and therefore plays a major role in maintaining basal Ca^2+^ concentrations in the cytoplasm and ER [46,47]. Several studies have demonstrated the expression of both STIM1 [48,49,50] and STIM2 [48] in the HT-22 cell line. It is of note that STIM1 and CaM interact with ORAI1 to induce the Ca^2+^-dependent inactivation of Ca^2+^ release-activated channels [51]. The binding of calcium-bound calmodulin (Ca^2+^-CaM) to the core region of the activated STIM1 facilitates a slow Ca^2+^-dependent inactivation after the ORAI1 channel activation [52].

On these grounds, the main goals of this work were: (i) to set up the experimental conditions for the internalization of an amount of Aβ(1-42) oligomers lower than the CaM content of the immortalized mouse hippocampal cell line HT-22, and to study its subcellular location, (ii) to experimentally assess if the dysregulation of the SOCE in the HT-22 cells by internalized Aβ(1-42) is an early event that takes place before the sustained alteration of cytosolic Ca^2+^ homeostasis, and (iii) to identify the main targets of internalized Aβ(1-42) associated with the SOCE components.

## 2. Results

### 2.1. Aβ(1-42) Internalizes Inside HT-22 Cells after a Short Period of Incubation and Co-Localizes with ER

**Figure 1:**
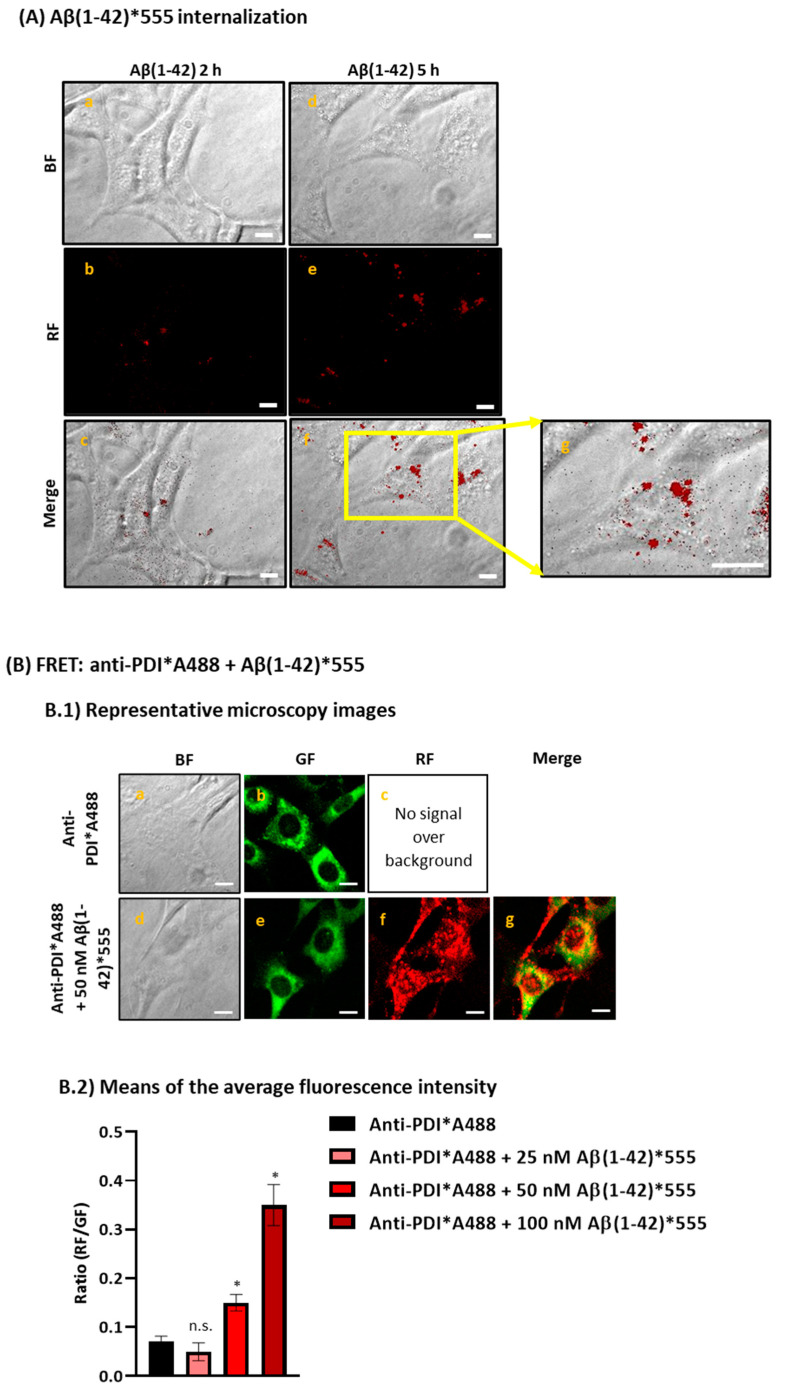
Internalization of Aβ(1-42)*555 after 2 h and 5 h incubation in living HT-22 cells and extensive FRET between anti-PDI and Aβ(1-42)*555 in fixed and permeabilized HT-22 cells. Panel (**A**): HT-22 cells were incubated with 2 μM Aβ(1-42) (1.8 μM of unlabelled Aβ(1-42) plus 0.2 μM of Aβ(1-42)*555) for 2 h and 5 h at 37 °C and 5% CO_2_. Figure 2A(a–c) shows representative images of bright field (BF), red fluorescence (RF), and Merge (BF plus RF) of HT-22 incubated for 2 h with Aβ(1-42). Figure 2A(d–f) shows representative images of BF, RF, and merge of HT-22 cells incubated for 5 h with Aβ(1-42). Figure 2A(g) shows a focalized zoom of Figure 2A(f) to highlight the subcellular distribution of Aβ(1-42)*555 near the perinuclear region of HT-22 soma. Scale bar inserted in fluorescence microscopy images = 20 μm. Panel (**B**): high co-localization between anti-PDI and Aβ(1-42)*555 as shown by the representative fluorescence microscopy images of HT-22 stained with anti-PDI/IgG-Alexa488 (PDI*A488, a–c) or with anti-PDI/IgG-Alexa488 plus 50 nM Aβ(1-42)*555 (PDI*A488/Aβ(1-42)*555, d–g) (**B.1**) and by the ratio of red/green fluorescence intensity per pixel (RF/GF) obtained from the analysis of fluorescence intensity data of HT-22 stained with anti-PDI/IgG-Alexa488 only (PDI*A488) and double stained with anti-PDI*IgG-Alexa488 plus 25, 50, or 100 nM Aβ(1-42)*555 (**B.2**). The results shown are the mean ± SEM (*) *p* < 0.05, (i.e., statistically significant relative to the control); n.s.—non-significant. Scale bar inserted in fluorescence microscopy images = 20 μm.

We evaluated the subcellular distribution of internalized Aβ(1-42) and measured the amount of Aβ(1-42) that is internalized inside living HT-22 cells by incubating the cells with 2 μM Aβ(1-42) for 2 h and 5 h. The representative microscopy images in Figure 1 (panel A) clearly show the internalization of human Aβ(1-42)-HiLyte™-Fluor555 (Aβ(1-42)*555) inside living HT-22 cells. The direct observation of the images demonstrated that after 2 h of incubation, Aβ(1-42) is distributed through all the cytoplasm, with focalized points near the nuclear region (Figure 1c, panel A), while after 5 h of incubation (Figure 1f–g, panel A), the subcellular distribution of Aβ(1-42)*555 is mainly near the perinuclear region. These results demonstrated that: (i) the Aβ(1-42) added to the extracellular medium is being internalized inside living HT-22 cells in a short period of incubation, and (ii) probably Aβ(1-42) is interacting with cytoplasmatic organelles, like the ER, mitochondria, and/or lysosomes, as it has been shown in other studies that intracellular Aβ interacts and modulates the functions of these organelles [19,20,21,22,53].

The concentration of internalized Aβ(1-42)*555 was calculated with a calibration curve obtained with different concentrations of Aβ(1-42)*555 added to the extracellular medium of fixed and permeabilized HT-22 cells (see Supplementary Figure S1). We calculated a concentration of 62 ± 11 nM and 135 ± 15 nM of internalized Aβ(1-42) monomers, after 2 h and 5 h of incubation in the HT-22 cells, respectively, assuming that there was not a significant difference between the rate of internalization of Aβ(1-42)*555 and unlabeled Aβ(1-42). The content of CaM in HT-22 cells has been measured by Western blotting (see Supplementary Figure S2). The analysis of the results obtained yields 0.92 ± 0.1 ng CaM/μg protein of HT-22 cells lysate. From this result we calculate a CaM concentration of 47 ± 5 μM, using the mass/volume ratio of 1,15 g/mL measured for hippocampal neurons [54]. Therefore, after 2 and 5 h of incubation, the amount of internalized Aβ(1-42) monomers is still much lower than the content of CaM in HT-22 cells.

To evaluate if internalized Aβ(1-42) is co-localizing with the ER, we used an antibody against protein disulfide isomerase (PDI), because this protein is one of the most abundant proteins in the ER and plays a major role as a molecular chaperone by catalyzing disulfide bond oxidation, reduction, and isomerization [55]. We used the FRET technique to assess the proximity between Aβ(1-42)*555 and the complex anti-PDI + AlexaFluor488-labeled anti-IgG antibody (anti-PDI*A488). First, we stained HT-22 cells with increasing concentrations of Aβ(1-42)*555 (25–100 nM), as described in the Materials and Methods section, aiming to perform the measurements with the lowest Aβ(1-42)*555 concentrations to highlight only the subcellular location of the high-affinity binding sites for Aβ(1-42). As can be seen in Supplementary Figure S3, the RF intensity increases as the Aβ(1-42)*555 concentration increases, and also the 3D surface plots highlight once more the subcellular distribution of Aβ(1-42)*555, which is more focalized near the HT-22 nucleus, as we demonstrated before in HT-22 living cells (Figure 1A). Then, to experimentally assess the occurrence of FRET between Anti-PDI*A488 and Aβ(1-42)*555, we selected the concentrations of 25, 50, and 100 nM of Aβ(1-42)*555. Figure 1, panel B.1, shows the representative microscopy images of HT-22 stained with anti-PDI*A488 in the absence and presence of 50 nM Aβ(1-42)*555. The direct observation of the images allows us to visualize a decrease in the GF intensity after the addition of 50 nM Aβ(1-42)*555 to the extracellular medium (Figure 1, B.1e), compared with cells stained with anti-PDI*A488 (Figure 1, B.1b) only, indicating the existence of FRET between this FRET pair. Furthermore, the merged image (Figure 1, B.1g) highlights the existence of punctate colocalization between anti-PDI*A488 and 50 nM Aβ(1-42)*555 in the neuronal soma, as shown by the yellow–orange pixels. In addition, the quantitative analysis (Figure 1, B.2) also demonstrates a high co-localization within the FRET distance between anti-PDI*A488 and Aβ(1-42)*555, as indicated by the 2.2-fold and 5.2-fold increase in the red/green fluorescence intensity ratio in the presence of 50 and 100 nM of Aβ(1-42)*555, respectively, after the subtraction of the red intensity by the direct excitation of Aβ(1-42)*555. On the other hand, there is no co-localization within the FRET distance between anti-PDI and 25 nM of Aβ(1-42)*555, as the same ratio values were calculated for anti-PDI in the absence or presence of 25 nM Aβ(1-42)*555. 

### 2.2. Aβ(1-42) Inhibits SOCE Activity after a Short Period of Incubation

**Figure 2:**
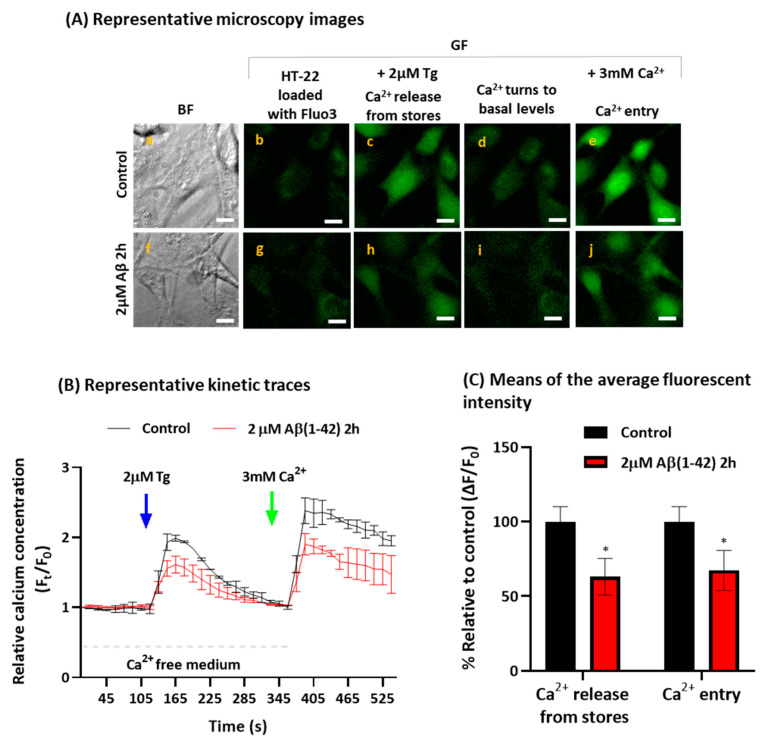
SOCE inhibition by Aβ(1-42) in HT-22 cells after 2 h of incubation. Untreated (control) and treated HT-22 cells incubated with 2 μM Aβ(1-42) for 2 h at 37 °C and 5% CO_2_ were loaded with 5 μM Fluo3-AM and 0.025% Pluronic^®^ F-127 for 1 h to experimentally evaluate Ca^2+^ imaging of SOCE. Panel (**A**): representative microscopy images of Fluo3-loaded untreated HT-22 (control) and cells treated with 2 μM Aβ(1-42) 2 h, acquired during SOCE experiments. (a,f) Bright field images (BF) of the fields selected for fluorescence images of Fluo3-loaded cells obtained before addition of 2 μM Tg (b,g), at the peak fluorescence after the addition of Tg (c,h), after completion of the Ca^2+^ release from the ER (d,i), and at the peak fluorescence after the addition of 3 mM Ca^2+^ (e,j). Scale bar = 20 μm. Panel (**B**): representative kinetic traces of untreated HT-22 cells (black trace) and HT-22 cells incubated with 2 μM Aβ(1-42) for 2 h (red trace) after the addition of 2 μM Tg (indicated by the blue arrow) for Ca^2+^ release from stores and after addition of 3 mM Ca^2+^ to evaluate the extension of Ca^2+^ entry in HT-22 cells through the plasma membrane (indicated by the green arrow). Panel (**C**): means of the average fluorescent intensity (ΔF/F_0_) relatively to control, after addition of Tg (Ca^2+^ release from stores) or after addition of Ca^2+^ (Ca^2+^ entry). Results show that Aβ(1-42) inhibits 37 ± 7% and 32 ± 6% of Ca^2+^ release from stores and Ca^2+^ entry, respectively, compared with control. Data are presented as the mean ± SEM of experiments done at least in 10 Petri plates in 5 independent assays (n > 80 cells, * *p* < 0.05, relatively to each control).

Since the internalized Aβ(1-42) interacts directly with the ER in HT-22 cells (Figure 1B), we have evaluated if Aβ(1-42) modulates the activity of the SOCE mechanism through a quantitative analysis of Ca^2+^ fluorescence imaging, using the Ca^2+^ indicator Fluo-3-pentaacetoxymethyl ester (Fluo3-AM). Figure 2A shows the representative microscopy images of Fluo3-loaded untreated and treated cells with 2 µM Aβ(1-42) for 2 h, before and after the addition of the SERCA blocker thapsigargin (Tg), and after the addition of Ca^2+^ (3 mM) to the extracellular medium. The direct observation of the images reveals that the cells treated with Aβ(1-42) are inhibiting SOCE activity, as seen by the decrease in the green fluorescence (GF) intensity in cells treated with Aβ(1-42), compared with the control group. The quantitative analysis (Figure 2B), shown by the representative kinetic traces of the SOCE experiments in untreated HT-22 cells (control, black trace) or cells treated with 2 μM Aβ(1-42) for 2 h (red trace), demonstrates a significant decrease in SOCE activity after HT-22 treatment with Aβ(1-42). These results showed the inhibition of both the Ca^2+^ release from the stores after the Tg addition, and the Ca^2+^ entry through the plasma membrane after the Ca^2+^ addition of 37 ± 7% and 32 ± 6%, respectively, compared with the control (Figure 2C). Controls performed with 2 μM of scrambled Aβ(1-42) for 2 h did not induce alterations in the SOCE mechanism, compared with untreated cells (control), as can be seen in Supplementary Figure S4.

**Figure 3:**
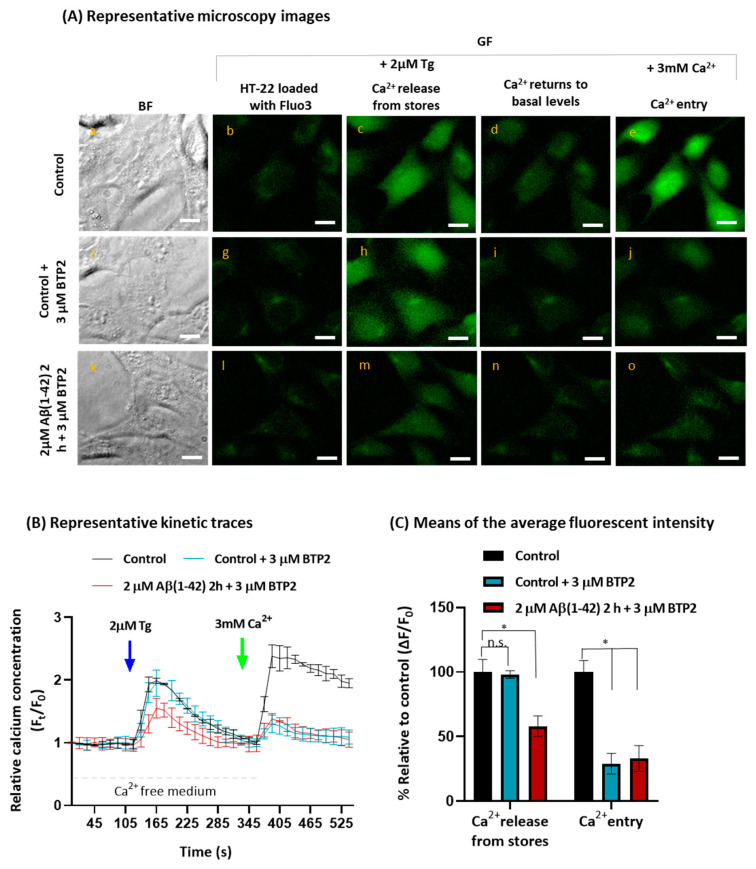
Effect of the SOCE inhibitor BTP2 in untreated (control) and treated HT-22 cells with Aβ(1-42). Untreated HT-22 cells (control) and treated cells with 2 μM Aβ(1-42) for 2 h were incubated with 3 μM of BTP2 for 15 min at 37 °C. Panel (**A**): representative fluorescence microscopy images of untreated HT-22 (control), cells with 3 μM BTP2, and cells treated with Aβ(1-42) plus 3 μM BTP2. (a,f,k) Bright field images (BF) of the fields selected for fluorescence images of Fluo3-loaded cells obtained before addition of 2 μM Tg (b,g,l), at the peak fluorescence after the addition of Tg (c,h,m), after completion of the Ca^2+^ release from the ER (d,i,n), and at the peak fluorescence after the addition of 3 mM Ca^2+^ (e,j,o). Scale bar = 20 μm. Panel (**B**): representative kinetic traces of HT-22 control with (blue trace line) or without BTP2 (black trace line) and HT-22 cells incubated with 2 μM Aβ(1-42) for 2 h plus BTP2 (red trace line) after addition of 2 μM Tg (indicated by the blue arrow) for Ca^2+^ release from stores, and addition of 3 mM Ca^2+^ to evaluate the extension of Ca^2+^ entry in HT-22 cells (indicated by the green arrow). Panel (**C**): means of the average fluorescent intensity (ΔF/F_0_) represented by percentage (%), relative to control cells. BTP2 does not elicit ER Ca^2+^ depletion but inhibits the Ca^2+^ influx in 71 ± 8% in control cells and HT-22 cells incubated with 2 μM Aβ(1-42) for 2 h, compared with the respective controls. Data are presented as the mean ± SEM of experiments done at least in 10 Petri plates in 5 independent assays (n > 80 cells for each condition) (* *p* < 0.05, relatively to each control). n.s.—non-significant.

As a positive control, we tested the STIM1-mediated SOCE inhibitor N-[4-[3,5-bis(trifluoromethyl)pyrazol-1-yl]phenyl]-4-methylthiadiazole-5-carboxamide (BTP2), which is considered a selective inhibitor because it does not affect Ca^2+^ signaling through the ER or mitochondria or other channel activities such as voltage-operated Ca^2+^ channels or K+ channels in T-cells [56,57]. We added 3 μM BTP2 to the HT-22 culture medium 15 min before the end of the incubation with Fluo3-AM (control cells), or before the end of the incubation for 2 h with Fluo3-AM plus 2 μM Aβ(1-42) in treated cells, and the results are shown in Figure 3. Figure 3A shows the representative microscopy images of Fluo3-loaded untreated and treated cells with 2 µM Aβ(1-42) for 2 h in the presence of BTP2. Both the representative images (Figure 3A) and the quantitative analysis (Figure 3B,C) shows that there is no significative difference in the Ca^2+^ released from the stores after the addition of Tg between the control cells in the absence (black trace, Figure 3B) and in the presence of 3 μM BTP2 (blue trace, Figure 3B), which are in a good agreement with the expected results, because BTP2 does not affect Ca^2+^ signaling through the ER, as mentioned before. On the other hand, the entry of Ca^2+^ through the plasma membrane decreases 71 ± 8% in the control cells incubated with BTP2, relative to the control without BTP2, which confirms that this Ca^2+^ influx is largely mediated by STIM1. Concerning the results obtained with HT-22 cells incubated with Aβ(1-42), there is no statistically significant difference in Ca^2+^ released after the addition of Tg from stores in HT-22 cells treated with 2 μM Aβ(1-42) only, 37 ± 7% inhibition (Figure 2C), and cells treated with both 2 μM Aβ(1-42) and 3 μM BTP2, 42 ± 6% inhibition (Figure 3C). Regarding the Ca^2+^ entry, the results show a two-fold decrease in Ca^2+^ influx after the HT-22 incubation with 2 μM Aβ(1-42) for 2 h plus 3 μM BTP2, a decrease that is the same as that observed with the HT-22 cells incubated with Aβ(1-42) only. These results strongly suggested that Aβ(1-42) is partially inhibiting the SOCE through the inhibition of the STIM1-mediated Ca^2+^ entry.

### 2.3. STIM1 Is a Target for Aβ(1-42)

**Figure 4:**
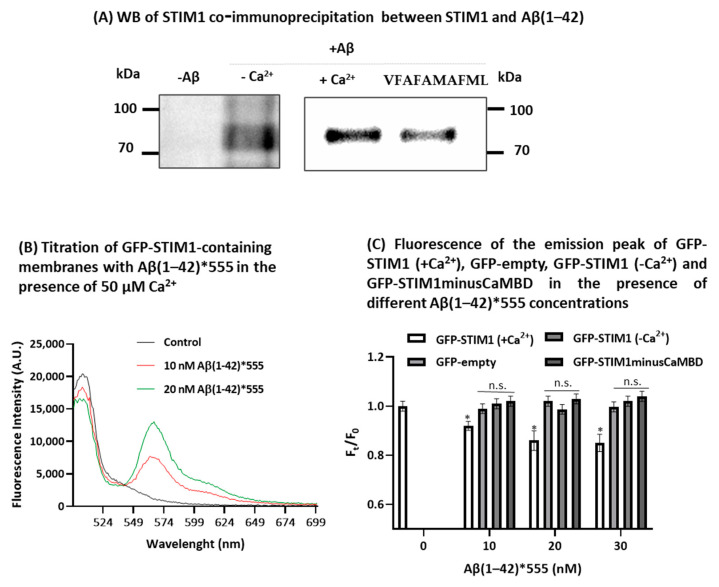
STIM1 is a target for Aβ(1-42). Panel (**A**): Western blots (WB) of STIM1 (anti-STIM1) after co-immunoprecipitation assay with mouse anti-Aβ antibody in the absence and presence of Ca^2+^, and in the presence of Ca^2+^ plus 1 μM of the peptide antagonist VFAFAMAFML, as described in the Materials and Methods section. Panel (**B**): titration of the fluorescence of STIM1-GFP in Ca^2+^ medium [STIM1-GFP (+Ca^2+^)] with different concentrations of Aβ(1-42)*555, using 10 μg/mL of HEK293 cell lysate containing STIM1-GFP in the absence (black trace) and after the addition of 10 nM (red trace) and 20 nM (green trace) of Aβ(1-42)*555. Fluorescence spectra were recorded at room temperature in buffer 50 mM N-[2-hydroxyethyl] piperazine-N′-[2-ethanesulfonic acid] (HEPES), 100 mM KCl, 2 mM MgCl_2_, and 50 μM CaCl_2_ (pH 7.05). The spectra were acquired between 20 and 30 min after the addition of the indicated Aβ(1-42)*555 concentration, with an excitation wavelength of 488 nm, and excitation and emission slits of 2 nm. Panel (**C**): fluorescence of the emission peak of STIM1-GFP(+Ca^2+^), GFP-empty, STIM1-GFP(-Ca^2+^), and STIM1-GFPminusCaMBD, in the presence of different Aβ(1-42)*555 concentrations. F and F_0_ are the fluorescence in the presence and the absence of Aβ(1-42)*555, respectively. The results shown are the means of triplicate experiments ± SEM (*) *p* < 0.05; n.s.—not statistically significant.

To understand the possible mechanism underlying the SOCE inhibition induced by Aβ(1-42), we assessed the interaction between Aβ(1-42) and STIM1, using (i) the co-immunoprecipitation method to evaluate the formation of STIM1-Aβ(1-42) complexes and (ii) the fluorescence resonance energy transfer (FRET) analysis between the STIM1-green fluorescent protein construct (STIM1-GFP) and Aβ(1-42)*555. The Western blots of Figure 4A reveal the formation of STIM1-Aβ complexes in HT-22 cell lysates, after co-immunoprecipitation with the anti-Aβ(1-42) antibody and in the presence of 250 nM Aβ(1-42), demonstrating that STIM1 is a target molecule of Aβ(1-42). Furthermore, in the presence of the peptide VFAFAMAFML (amidated-C-terminus amino acid), an antagonist of the CaM:Aβ(1-42) complexation designed and experimentally tested in our recent publication [8], the co-immunoprecipitation of STIM1 is reduced between 40 and 50%. Thus, this result pointed out that CaM potentiates the binding of Aβ(1-42) to STIM1. 

**Figure 5:**
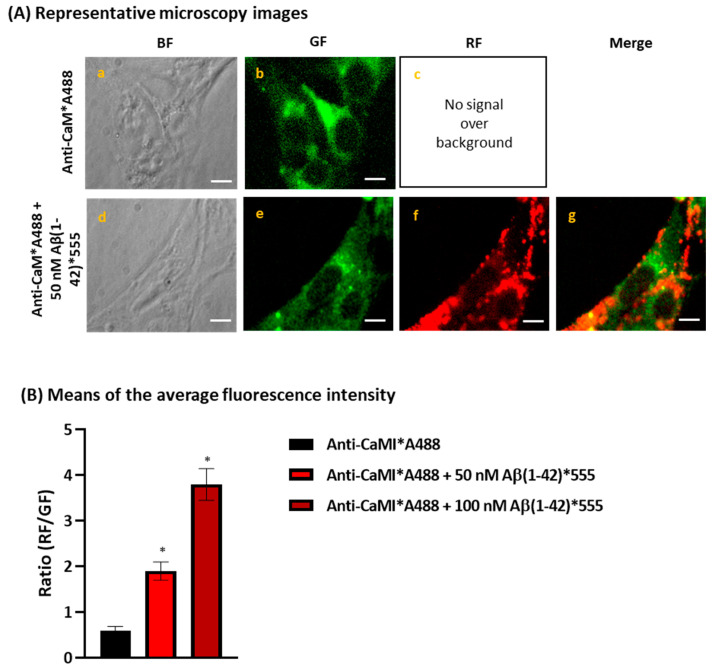
Extensive FRET between CaM and Aβ(1-42)*555. Panel (**A**): representative fluorescence microscopy images of HT-22 stained with anti-CaM/IgG-Alexa488 (CaM*A488, **a**–**c**) or with anti-CaM/IgG-Alexa488 plus 50 nM Aβ(1-42)*555 (CaM*A488/Aβ(1-42)*555, **d**–**g**). BF, GF, and RF images are shown for each of the selected fields, and the orange–yellow areas (merge image, **g**) point out for higher intensity FRET pixels. The exposure time for GF and RF images was 0.4 s. Scale bar inserted in fluorescence microscopy images = 20 μm. Panel (**B**): ratio of red/green fluorescence intensity per pixel (RF/GF) obtained from the analysis of fluorescence intensity data of HT-22 stained with anti-CaM/IgG-Alexa488 only (CaM*A488) and double stained with anti-CaM*IgG-Alexa488 plus 50 or 100 nM of Aβ(1-42)*555. The results shown are the mean ± SEM (*) *p* < 0.05 (i.e., statistically significant relative to the control).

Next, we assessed the binding of Aβ(1-42) with STIM1 using FRET between STIM1-GFP and Aβ(1-42)*555. To this end, we have prepared, as indicated in the Materials and Methods, samples of the membrane fraction of hypotonic lysates of HEK293 cells expressing STIM1-GFP. The effect of the titration of STIM1-GFP-containing membranes with Aβ(1-42)*555 in the presence of 50 μM Ca^2+^ are shown in Figure 4B. In Figure 4C are the plotted means ± SEM of the results obtained in the experimental triplicates using the samples of the membrane fractions of the HEK293 cells expressing STIM1-GFP in the presence of Ca^2+^, and also in the cells stably expressing a green fluorescent protein (GFP), i.e., transfected for the expression of the GFP-empty vector. The results of Figure 5B,C show that in a Ca^2+^ medium, Aβ(1-42)*555 produces a 14 ± 4% quenching of STIM1-GFP fluorescence at saturation by FRET to Aβ(1-42)*555, but not of GFP-empty used as the control (Figure 4C), excluding the possibility that the observed quenching was due to a direct interaction of Aβ(1-42)*555 with the GFP protein attached to STIM1. In addition, the results shown in Figure 5C yield an IC50 value between 5 and 10 nM Aβ(1-42)*555 for the quenching of STIM1-GFP fluorescence. Using the R_0_ value for the FRET pair GFP (donor)/Aβ(1-42)*555 (acceptor) obtained in this work and given in the Materials and Methods section, we can calculate that Aβ(1-42)*555 binds to a site that is between 6 and 7 nm distance from the GFP fluorochrome in STIM1-GFP, assuming that about 50% of the membranes are inverted concerning the right-side out orientation. 

Since CaM binding to STIM1 produces the inhibition of the SOCE [51,52], and in previous works we showed that CaM is a major target protein for Aβ(1-42) [8,9], we have performed these experiments also in the absence of Ca^2+^ as well as using a membrane fraction from cells stably expressing STIM1-GFP with a deletion of the amino acids 235–442 belonging to the cytosolic domain, which lacks the amino acids 368–391 of the CaM-binding domain in the core region of STIM1 (STIM1-GFPminusCaMBD). The results (Figure 4C) showed no FRET between GFP-STIM1 and Aβ(1-42)*555 in the Ca^2+^-free medium, demonstrating that Ca^2+^ plays a key role in the Aβ(1-42) and STIM1 complexation. Furthermore, the results obtained with the STIM1-GFPminusCaMBD revealed also the absence of quenching by Aβ(1-42)*555 up to 30 nM in the presence of 50 μM Ca^2+^. To further evaluate the hypothesis that the Aβ(1-42)-CaM complexation can mediate the binding between STIM1 and Aβ(1-42), we experimentally assessed if CaM and Aβ(1-42) are also within FRET distance in fixed and permeabilized HT-22 cells in a culture by using anti-CaM conjugated with the secondary antibody IgG Alexa488 (anti-CaM*A488) in the absence and presence of 50 and 100 nM of Aβ(1-42)*555. The representative fluorescence microscopy images of Figure 5A show an extensive co-localization between anti-CaM*A488 and 50 nM Aβ(1-42)*555, as can be seen by the large decrease in the GF after the addition of Aβ(1-42)*555 to the extracellular medium (compare images e and b of Figure 5A). Moreover, the orange–yellow pixels presented in the merge image g of Figure 5A reveal the high colocalization of Aβ(1-42)*555 with anti-CaM. The quantitative analysis of these images (Figure 5B) also indicates a large increase in the red/green fluorescence intensity ratio in the presence of 50 and 100 nM of Aβ(1-42)*555, after a subtraction of the red intensity by the direct excitation of Aβ(1-42)*555, yielding a 3.4-fold and 6.9-fold increase, respectively. Therefore, these results point out that anti-CaM*A488 and 50 nM Aβ(1-42)*555 are within FRET distance, and that CaM is, also, a major binding target protein for Aβ(1-42) in HT-22 cells.

### 2.4. Aβ(1-42) Induces a Low to Moderate Increase in Reactive Oxygen Species (ROS) Production without Altering the Mitochondria Membrane Potential after a Short Period of Incubation in HT-22 Cells

**Figure 6:**
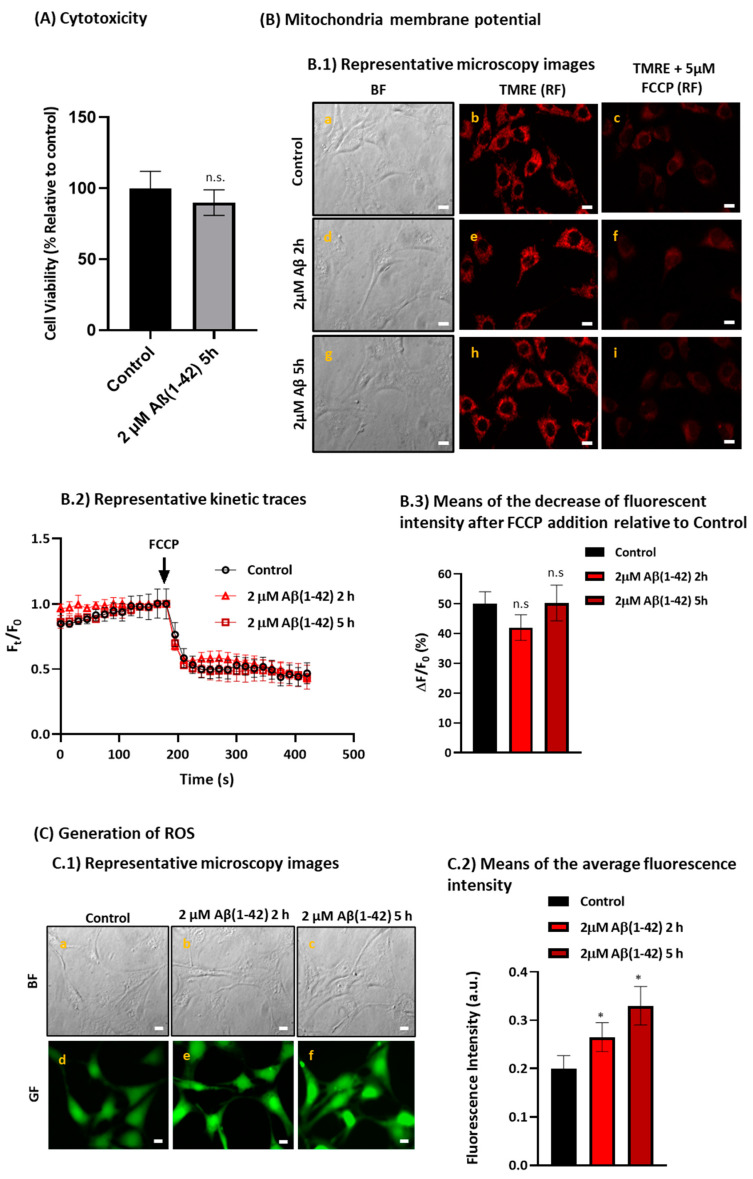
Treatment of HT-22 cells with 2 µM Aβ(1-42) up to 5 h incubation does not induce cytotoxicity nor alter the mitochondria membrane potential in the HT-22 cell model but induces a low to moderate increase in ROS production. Untreated cells (control) and HT-22 cells incubated with 2 μM Aβ(1-42) for 2 h and/or 5 h at 37 °C and 5% CO_2_ were evaluated for (i) cell viability using the MTT assay Panel (**A**); (ii) mitochondria membrane potential using the TMRE assay Panel (**B**); and (iii) ROS generation by monitoring the H_2_DCF-DA oxidation to the highly fluorescent dichlorofluorescein Panel (**C**). Panel (**A**): the 10% loss of cell viability of HT-22 cells after incubation with 2 μM Aβ(1-42) for 5 h is statistically non-significant (*p* > 0.05). Cell viability is expressed as a percentage relative to the control. Panel (**B**): (**B.1**) the mitochondria membrane potential is not statistically significant affected by Aβ(1-42) in a short period of time (*p* > 0.05), as showed by the representative microscopy images. BF are the bright field images (a,d,g) of the selected fluorescence microscopy images acquired after the cell staining with TMRE before (b,e,h), and 150 s after the addition of FCCP (c,f,i), see the kinetic traces of panel B.2. (**B.2**) Representative kinetic traces of the average fluorescence intensity (Ft/F_0_) recorded over time. (**B.3**) Means of the decrease of fluorescent intensity before and after the addition of 5 μM FCCP (ΔF/F_0_) relative to the Control. Panel (**C**): Aβ(1-42) induces a low to moderate increase in ROS production after 2 h and 5 h incubation in HT-22 cells, as indicated by the representative green fluorescence (GF) microscopy images of untreated HT-22 cells (d) and treated cells incubated with 2 μM Aβ(1-42) for 2 h and 5 h after addition of 10 μM H_2_DCFDA (e and f, respectively) (**C.1**), and by the means of the average fluorescence per pixel in HT-22 cells (**C.2**). BF images a, b and c are the bright field images of the fluorescence images d, e and f, respectively. The results show a 1.3-fold and 1.7-fold increase in ROS generation in HT-22 cells treated with 2 μM Aβ(1-42) for 2 h and 5 h, respectively, compared with untreated cells (control) (* *p* < 0.05). The results presented are the mean ± standard error of the mean (SEM) of experiments done at least in 8 Petri plates in 4 independent assays (n > 60 cells). Fluorescence intensity in arbitrary units (a.u.); n.s.—not significant. Scale bar = 20μm.

The putative cytotoxicity of Aβ(1-42) was evaluated in the HT-22 cell line and the cell viability was measured using the 3-(4,5-dimethylthiazol-2-yl)-2,5-diphenyltetrazolium bromide (MTT) assay. The results shown in Figure 6 (panel A) demonstrate there is not a significant loss of cell viability (*p* > 0.05) after 5 h of incubation with 2 μM Aβ(1-42), compared with untread cells (control group). Furthermore, we evaluated if the mitochondria membrane potential was altered by Aβ(1-42) in the HT-22 cells after a short period of incubation, using the tetramethylrhodamine ethyl ester (TMRE) assay. As a positive control of complete mitochondrial depolarization, we added to the extracellular medium of TMRE-loaded HT-22 cells 5 μM of trifluoromethoxy carbonylcyanide phenylhydrazone (FCCP), a potent mitochondrial oxidative phosphorylation uncoupler. The direct observation of the representative microscopy images (Figure 6, panel B.1) and the representative kinetic traces of the average fluorescence intensity per pixel (Ft/F_0_) (Figure 6, panel B.2) show a large decrease in the mitochondria membrane potential after the addition of FCCP, as expected. The difference in the average fluorescent intensity before and after the addition of FCCP between the control group and HT-22 cells treated with Aβ(1-42) was not statistically significant (*p* > 0.05) (Figure 6, panel B.3), indicating that Aβ(1-42) is not altering the mitochondria membrane potential in HT-22 cells in the conditions used in this work. 

It has been reported that oxidative stress plays a fundamental role in AD pathology, therefore, we evaluated the generation of intracellular ROS and measured the reduced glutathione (GSH) levels through the monitoring of 2′,7′-dichlorodihydrofluorescein diacetate (H_2_DCF-DA) oxidation and by measuring the conversion of monochlorobimane (MCB) to the fluorescent glutathione conjugate (GS-MCB), respectively. The results in Figure 6, panel C indicate a 1.3- and 1.7-fold increase in ROS production after 2 h and 5 h of incubation with 2 µM Aβ(1-42), respectively, compared with the untreated cells. However, the GSH levels in the HT-22 cells treated with 2 µM Aβ(1-42) after 2 h or 5 h of incubation are not significantly different from that of the untreated cells (see Supplementary Figure S5). Taken together, these results indicate that Aβ(1-42) is inducing a low increase in ROS production after a short period of incubation in HT-22 cells. 

## 3. Discussion

In this work, we aimed to identify the targets relevant for intracellular Ca^2+^ homeostasis primarily modulated by concentrations of internalized Aβ(1-42) which were lower than the CaM concentration in HT-22 cell cultures, with less than 25% of cells presenting the cholinergic phenotype. We found that a short treatment of in vitro differentiated HT-22 cells with 2 μM of Aβ(1-42) small oligomers, largely dimers, yielded approximately 62 ± 11 nM and 135 ± 15 nM of internalized Aβ(1-42), after 2 h and 5 h of incubation, respectively. The content of CaM in HT-22 cells determined by Western blotting in this work is 0.92 ± 0.1 ng CaM/μg protein of lysate, which yields a CaM concentration of 47 ± 5 μM using the value of 1.15 g/mL reported for the mass/volume ratio of neurons [54]. Thus, our results allow us to conclude that after 2 and 5 h of incubation, the concentration of internalized Aβ(1-42) monomers is still much lower than the concentration of CaM in HT-22 cells. In a previous work [6], we showed that mature cerebellar granule neurons internalized about 193 ± 21 nM of Aβ(1-42) only after 2 h of incubation, i.e., three times more than HT-22 cells. Increasing evidence suggests that Aβ peptides are internalized in neuronal cells through different pathways, which also depend on the cell type [22,58,59]. It has been reported that neuronal lipid rafts microdomains play a major role in Aβ formation/oligomerization and Aβ uptake by neurons, reviewed in [60], and we showed in a previous study that mature cerebellar granule neurons express high levels of lipid rafts markers like cholera toxin B binding sites, caveolin-1, flotillin, and HRas [61,62]. In contrast, we found in this work that HT-22 cells were not stained with cholera toxin B-Alexa488, demonstrating the lack of lipid rafts in this mouse hippocampal cell line HT-22 [63]. The lipid rafts-mediated endocytosis does not require the organization of a complex clathrin coat and, therefore, takes place faster than clathrin-mediated endocytosis [64].

Our results showed that the treatment of the HT-22 cells with 2 μM Aβ(1-42) small oligomers for up to 5 h does not elicit a significant loss of cell viability, nor of mitochondrial membrane potential, and only a low to moderate increase in the intracellular oxidative stress without a significant decrease in the intracellular reduced glutathione. In another published study performed on HT-22 cells, it was concluded that exogenous treatment of Aβ(1-42) induced mitochondrial impairment, but it is important to note that the HT-22 cells were incubated with 5 μM of Aβ(1-42), i.e., 2.5-fold the concentration used in this work, and the mitochondrial dysfunction was evaluated 6 h after Aβ(1-42) the incubation of the HT-22 cells. 

Using fluorescence microscopy imaging, we demonstrated that after 2 h of incubation with 2 µM Aβ(1-42), internalized Aβ(1-42) is distributed across all the HT-22 cytoplasm, with some focalized points near the nuclear region. Later on, after 5 h of incubation, internalized Aβ(1-42) is showing a subcellular distribution, mainly near the perinuclear region. These results are in agreement with other studies, demonstrating that Aβ(1-40) and/or Aβ(1-42) peptides are predominantly distributed in the perinuclear region in differentiated PC12 cells and rat primary hippocampal neurons [58], and the neuroblastoma cell line SH-SY5Y [22,59]. The intracellular distribution of internalized Aβ(1-42) in HT-22 cells suggested that it is extensively bound to subcellular organelles. However, internalized Aβ(1-42)*555 displayed only a marginal co-localization with MitoTracker™ Green FM (Supplementary Figure S6), an experimental fact that is in good agreement with the lack of a significant change in the mitochondria membrane potential in HT-22 cells after 2 or 5 h of treatment with 2 μM Aβ(1-42). Next, we evaluated if Aβ(1-42) is co-localizing with the ER. To this end, we used PDI as the ER marker and evaluated the existence of FRET between a complex anti-PDI + AlexaFluor488-labeled anti-IgG antibody(anti-PDI*A488) and different concentrations of Aβ(1-42)*555. The results clearly showed an extensive co-localization between anti-PDI*A488 and Aβ(1-42)*555, as demonstrated by the decrease in the GF intensity after the addition of Aβ(1-42)*555 and by the increase in the red/green fluorescence intensity ratio in the presence of 50 and 100 nM of Aβ(1-42)*555. Thus, these results allowed us to conclude that the ER is a major target organelle in this concentration range of internalized Aβ(1-42)*555. It has been demonstrated that nitric oxide generated in AD induces the S-nitrosylation of PDI and inhibits its enzymatic activity, leading to an unfolded protein response, which induces the ER stress and may cause apoptosis of neuronal cells through S-nitrosylation and the downregulation of PDI in AD [65]. Thus, PDI has been seen as a potential target for AD therapy [66]. However, our measurements using the isomerase assay described in [67] showed that up to 200 nM Aβ(1-42) did not significantly inhibit the activity of PDI in HT-22 cell lysates [63].

It has been reported that the SOCE function is diminished in AD, resulting in the instability of dendric spines and enhanced amyloid genesis [27]. A large number of studies performed on familial AD have provided mechanistic insights regarding the altered SOCE function in AD [27]. Other studies have shown an attenuated Ca^2+^ entry in the skin fibroblasts from familial AD patients [29,30]. Furthermore, it has been demonstrated that STIM1 is cleaved by the presenilin 1-secretase, leading to the dysregulation of Ca^2+^ homeostasis in SH-SY5Y and fibroblasts from familial AD patients [31]. Additionally, we have reported that the levels of STIM1 expression in brain tissues of the medium frontal gyrus are decreased with the progression of neurodegeneration [68]. As internalized Aβ(1-42) is interacting with the ER in HT-22 cells, the possibility that SOCE activity could be a target for Aβ(1-42) deserved to be studied. Our results showed that incubation of HT-22 cells with 2 μM Aβ(1-42) for 2 h is affecting the Ca^2+^ signaling mechanism of SOCE in a dual mode, the partial depletion of the Ca^2+^ stores, and the partial inhibition of the Ca^2+^ influx through the plasma membrane elicited by the addition of Ca^2+^ to the extracellular medium after the Tg-induced Ca^2+^ release from the ER. HT-22 cells treated with Aβ(1-42) showed a 37 ± 7% decrease in the Tg-induced Ca^2+^ release from the stores, and a 32 ± 6% inhibition of the Ca^2+^ influx through the plasma membrane, compared with the control group. However, in the absence of the Tg, we did not detect an SOCE which was induced by Aβ(1-42), a result that is consistent with the lack of SERCA inhibition by amyloid peptides [69], suggesting that Aβ(1-42) has an overall negative impact on the neuronal response to transient ATP depletion stress. 

BTP2 is a cell-permeable pyrazole that acts as a potent blocker of STIM1-mediated SOCE. Therefore, we used BTP2 as a positive control of SOCE inhibition by incubating untreated and treated HT-22 cells with 3 μM BTP2 for 15 min. This treatment led to a significant decrease in the Ca^2+^ influx (71 ± 8%) after the addition of Ca^2+^ to the extracellular medium, without affecting the amount of Ca^2+^ released from the ER. These results suggested the possibility that STIM1 could be a protein target of internalized Aβ(1-42) in HT-22 cells. This possibility was confirmed using HT-22 lysates by co-immunoprecipitation with the anti-Aβ(1-42) antibody and in the presence of 250 nM Aβ(1-42). The co-immunoprecipitation of anti-STIM1 was assessed by Western blotting, demonstrating that STIM1 is a target molecule of Aβ(1-42) at the concentrations that this Aβ peptide reached in the HT-22 cells, after incubation for 2 h with 2 μM Aβ(1-42) added to the extracellular medium. The formation of STIM1-Aβ(1-42) complexes is also shown by the partial quenching of the fluorescence of STIM1-GFP expressed in HEK293 cells that is produced by nanomolar concentrations of Aβ(1-42)*555. Indeed, the titration of STIM1-GFP fluorescence with different concentrations of Aβ(1-42)*555 allows us to calculate a dissociation constant of Aβ(1-42)*555 from these complexes in the range of 5–10 nM. Additionally, the efficiency of FRET obtained in these experiments allows us to conclude that Aβ(1-42)*555 binds to a site located ≈ 6–7 nm from the donor dye of the GFP protein attached to the C-terminal of STIM1. Since the fluorochrome of the GFP protein is near the middle of its 4.2 nm long barrel-like structure, these results pointed out that the binding site of Aβ(1-42)*555 is located in the large cytosolic domain of STIM1, at a distance between 4 and 5 nm from the C-terminus amino acid of STIM1. Our results showed, also, that the interaction between Aβ(1-42)*555 and STIM1-GFP is calcium-dependent, since no significant quenching was observed in a Ca^2+^-free medium [STIM1-GFP(-Ca^2+^)], and that the peptide VFAFAMAFML (amidated-C-terminus amino acid), a peptide antagonist of the Aβ(1-42):CaM complexation [8], reduced between 40 and 50% of the co-immunoprecipitation of STIM1. Altogether, these results suggested the possibility that Aβ(1-42)*555 alone or complexed with CaM binds to the cytosolic calcium-CaM-binding domain of STIM1, which is an inhibitory site and involves several amino acids between 368 and 391 located at the SOAR domain of STIM1 [51], i.e., nearly 300 amino acids distant from the C-terminus amino acid of STIM1. To experimentally assess this hypothesis, we prepared a construct lacking the CaM-binding domain of STIM1 (STIM1-GFPminusCaMBD). Our results revealed that the removal of the CaMBD of STIM1 led to the loss of the STIM1-GFP fluorescence quenching elicited by nanomolar Aβ(1-42)*555 concentrations. In addition, our FRET-imaging results showed an extensive complexation between Aβ(1-42)-CaM in HT-22 cells at the concentrations of the internalized Aβ(1-42)*555 reached after incubation for 2 h with 2 μM Aβ(1-42), added to the extracellular medium. This result is in a good agreement with the results reported in our previous work with cerebellar granule neurons [6]. Although that deletion on STIM1 would have a large impact on the oligomerization of the STIM1 protein, and therefore further experimental studies will be required for the definition of the binding site of Aβ(1-42) in STIM1:CaM complexes, our results lend a strong support to the hypothesis that CaM potentiates the binding between STIM1 and Aβ(1-42).

**Figure 7:**
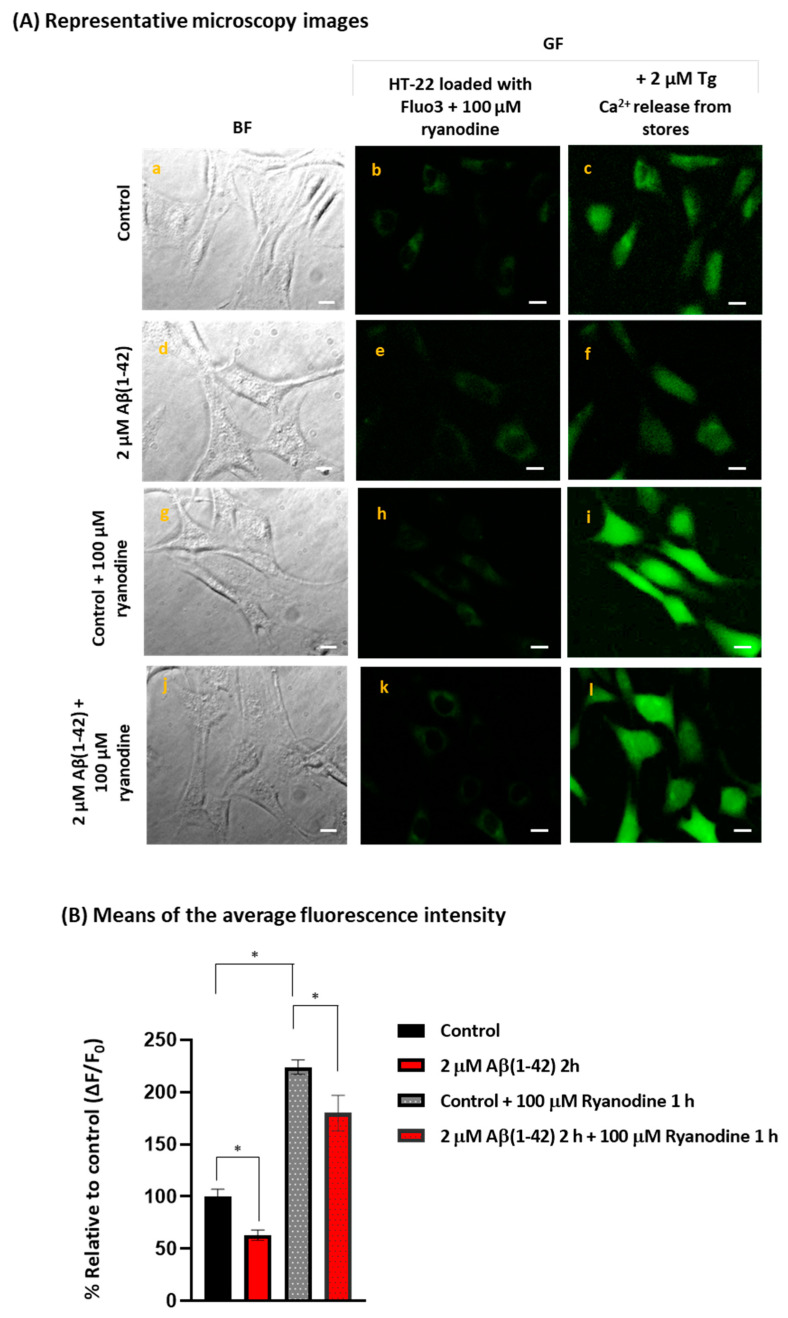
Aβ(1-42) partially stimulates RyR activity after 2 h of incubation in HT-22 cells. Untreated (control) and treated HT-22 cells with 2 μM Aβ(1-42) for 2 h were loaded with Fluo3-AM plus 0.025% Pluronic^®^ F-127 in the absence and presence of the RyR inhibitor ryanodine (100 μM) for 1 h at 37 °C. Panel (**A**): representative microscopy images of untreated (control) and cells treated with Aβ(1-42) in the absence and presence of 100 µM ryanodine, before and after the addition of 2 µM Tg to the extracellular Ca^2+^-free medium for Ca^2+^ release from the stores. Bright field images (a,d,g,j) of the fluorescence images of Fluo3-loaded cells acquired before the addition of 2 μM Tg (b,e,h,k), and peak fluorescence images after de addition of Tg (c,f,i,l). Scale bar = 20 µm. Panel (**B**): means of the average peak fluorescence intensity per pixel after addition of 2 μM Tg to the extracellular Ca^2+^-free MLocke’s K5 medium in untreated cells (control) and HT-22 treated with 2 μM Aβ(1-42), in the absence and presence of 100 μM ryanodine. The results shown are the mean ± SEM. (*) *p* < 0.05. The results indicated that ryanodine blocks the RyR, as demonstrated by the significant increase in Ca^2+^ release from stores (2.2-fold) in control cells incubated with the inhibitor, compared with control cells without ryanodine. Additionally, there is a decrease in Ca^2+^ release from stores (±20%) in HT-22 cells treated with Aβ(1-42) plus 100 μM ryanodine, compared with cells incubated only with ryanodine (control plus ryanodine), indicating that Aβ(1-42) partially stimulates the RyR activity.

Besides the inhibition of the SOCE-dependent Ca^2+^ influx through the plasma membrane, the incubation of HT-22 cells for 2 h with 2 μM Aβ(1-42) also induced a 37% decrease in the Ca^2+^ released from the stores. Indeed, the stimulation of the ER Ca^2+^-leak channels by this treatment of HT-22 cells with 2 μM Aβ(1-42) do not seem to impair intracellular Ca^2+^ homeostasis, since we have not observed a significant increase in the cytosolic Ca^2+^ concentration neither in Fura2-loaded nor in Fluo3-loaded HT-22 cells, up to 5 h of incubation with 2 μM Aβ(1-42) added to the extracellular medium (see the Supplementary Figure S7). This latter result shows that HT-22 cells can maintain the resting cytosolic Ca^2+^ homeostasis for several hours after treatment with 2 μM Aβ(1-42), suggesting that during this time, the systems that extrude calcium from the cytosol can fully compensate for the partial release of calcium from the ER. A decrease in the Ca^2+^ content of the ER was an expected result because it has been shown that Aβ(1-42) stimulates RyR-mediated Ca^2+^ release in hippocampal neurons in the culture [70], and, also, that Aβ(1-40) induced an early increase in the intracellular Ca^2+^ levels due to the release of Ca^2+^ from the ER through RyR and IP3R in cortical neurons [38]. In this work, we have experimentally assessed that the ER Ca^2+^ content of our HT22 cell cultures increases about 2.2-fold upon a blockade of the Ca^2+^ release through RyR by ryanodine (Figure 7), a highly selective RyR antagonist [71]. In addition, Figure 7 shows that the increase in Tg-induced Ca^2+^ released from the stores in HT-22 cells treated with 2 μM Aβ(1-42) for 2 h plus 100 μM ryanodine, is approximately three-fold higher compared with cells treated only with Aβ(1-42), a result that is consistent with a weak stimulation of RyR in HT-22 cells treated with 2 μM Aβ(1-42) for 2 h. 

In summary, we showed that Aβ(1-42) internalizes inside HT-22 cells in a sub-micromolar concentration after a short period of incubation and localizes mainly at the perinuclear region up to 5 h of incubation in HT-22 cells, before inducing a significant loss of cell viability. Additionally, Aβ(1-42) showed an extensive colocalization with the ER without affecting significantly the mitochondrial membrane potential. The modulation of the ER induced by Aβ(1-42) after 2 h of incubation caused a decrease in the SOCE mechanism through the modulation of STIM1, and the ER Ca^2+^-leak channels, which do not seem to be mediated by the ROS generation in HT-22 cells in the conditions used in this work.

## 4. Materials and Methods

### 4.1. Chemicals 

Human Aβ(1-42)-HiLyte™-Fluor555 (Aβ(1-42)*555) was obtained from AnaSpec (Freemont, CA, USA). Unlabeled Aβ(1-42) and scrambled Aβ(1-42) were synthesized and supplied by StabVida (Caparica, Portugal). Purified bovine brain CaM was purchased from Sigma-Aldrich (Madrid, Spain). Fluo-3-pentaacetoxymethyl ester (Fluo3-AM), Fura2 acetoxymethyl ester (Fura2-AM), and Pluronic^®^F-127 were obtained from Biotium (Hayward, CA, USA). Thapsigargin (Tg) was obtained from Sigma-Aldrich (Madrid, Spain) and BTP2 was from Merck Roche–Merck (Darmstadt, Germany). Ryanodine was purchased from Tocris (Bristol, UK). Tetramethylrhodamine ethyl ester (TMRE) and 2′,7′-dichlorodihydrofluorescein diacetate (H_2_DCFDA) were supplied by Invitrogen (Molecular Probes, Eugene, Oregon, USA). Xestospongin C (XeC) and Protein A/G PLUS-Agarose sc-2003 were purchased from Santa Cruz Biotechnology Inc. (Santa Cruz, CA, USA).

Primary antibodies: goat anti-protein disulfide isomerase (PDI) (sc17222) was supplied by Santa Cruz Biotechnology (Santa Cruz, CA, USA). Rabbit anti-CaM (anti-CaM) (Epitomics 1716-1) antibody was supplied by Abcam (Cambridge, UK). Anti-STIM1 was raised against the peptide encompassing residues 614–628 of STIM1, and its specificity has been described in previous works [72,73]. Monoclonal mouse anti-Aβ antibody (A8354) was purchased from Sigma-Aldrich (Madrid, Spain). Fluorescent-labeled secondary antibodies used to label the primary antibodies listed above were anti-rabbit IgG-Alexa488 (A11008) and anti-goat IgG-Alexa488 (A11055) from Invitrogen (Molecular Probes, Eugene, OR, USA). Anti-goat IgG-horseradish peroxidase was supplied by Sigma-Aldrich. Bio-Rad Clarity Western ECL substrate was purchased from Bio-Rad (Alcobendas, Madrid, Spain). 

All other reagents and chemicals were of analytical grade from Sigma-Aldrich (Madrid, Spain) or Roche–Merck (Darmstadt, Germany).

### 4.2. HT-22 Cell Culture 

The immortalized mouse hippocampal neuronal HT-22 cells were cultured in Dulbecco’s modified Eagle’s medium (DMEM)-high glucose, supplemented with 10% inactivated fetal bovine serum, 4 mM glutamine, 1 mM pyruvic acid, 100 U/mL penicillin, and 100 μg/mL streptomycin, and kept at 37 °C in a humidified atmosphere of 95% air/5% CO_2_ until reaching a 70–80% confluency in the T-flask. For all experiments, the HT-22 cells were seeded in 35 mm dishes at a density of 8 × 10^3^ cells/cm^2^ in the culture media described above and allowed to grow for 48 h at 37 °C and 5% CO_2_ before performing each experiment.

### 4.3. Cell Viability 

The HT-22 cells were incubated with 2 μM Aβ(1-42) for 5 h at 37 °C and 5% CO_2_ with gentle mixing. Then, cells were washed with 1 mL of MLocke’s K5 buffer (pH 7.4 at 37 °C): 4 mM of NaHCO_3_, 10 mM of Tricine, 5 mM of glucose, 2.3 mM of CaCl_2_, 1 mM of MgCl_2_, 154 mM of NaCl, and 5 mM of KCl. Cell viability was experimentally assessed by measuring the amount of colored formazan by the reduction of MTT, as in our previous works [6,61,74]. Untreated cells were regarded as controls (100% cell survival) and the cell survival ratio was expressed as the percentage of the control.

### 4.4. Mitochondrial Membrane Potential 

Mitochondrial depolarization was monitored with the fluorescent dye TMRE, as previously described, with small changes [74]. Briefly, the HT-22 cells were incubated with 2 μM of Aβ(1-42) for 2 h or 5 h at 37 °C and 5% CO_2_. Next, the untreated cells (control) and cells treated with Aβ(1-42) were washed once with MLocke’s K5 buffer and loaded with 100 nM of TMRE for 10 min at 37 °C and 5% CO_2_ and with continuous and gentle mixing. Then, the HT-22 culture dishes were placed in the thermostatic plate at 37 °C of the inverted epifluorescence microscope and the fluorescence kinetic was recorded at 556 nm excitation filter and a dichroic mirror of 580 nm with an emission filter of 590 nm and a time of exposure 0.01 s. To assess the level of the mitochondrial membrane depolarization, 5 μM of a potent mitochondrial oxidative phosphorylation uncoupler FCCP was added to the medium. The region of interest (ROI) tool of the HCImage software was used for the quantitative analysis. The average fluorescence intensity readings per pixel in the HT-22 cells were taken from several fields for a total number of 80 cells.

### 4.5. Measurement of Cellular Oxidative Stress 

For the detection of intracellular ROS levels, we measured the oxidation of H_2_DCFDA to the fluorescent compound dichlorofluorescein, as previously described, with minor modifications [74]. Briefly, the untreated HT-22 (control) and treated cells incubated with 2 μM of Aβ(1-42) for 2 h or 5 h at 37 °C and 5% CO_2_ were washed once with MLocke’s K5 buffer and placed in the thermostatically controlled plate at 37 °C of the Nikon Diaphot 300 inverted epifluorescence microscope. Then, 10 μM of H_2_DCFDA was added to the medium and the fluorescence intensity was recorded every 15 s for 10 min with an excitation filter of 470 nm, and 510 nm dichroic mirror/520 nm emission filter, using an exposure time of 0.2 s.

The cellular GSH level was monitored with MCB using fluorescence microscopy, as described in our previous work [73]. Images were acquired with a Nikon Diaphot 300 epifluorescence microscope, with an excitation filter of 380 nm.

### 4.6. Measurement of Internalized Aβ(1-42)*555 

The internalization of Aβ(1-42)*555 in the HT-22 cell culture was measured using a similar experimental approach as to the one described earlier [6], with small modifications. The amount of Aβ(1-42) internalized was estimated from the increase in the red fluorescence (RF) intensity in the HT-22 cells at different times of incubation (2 h and 5 h), at 37 °C and 5% CO_2_, with a total concentration of 2 μM of Aβ(1-42) (1.6 μM of unlabeled Aβ(1-42) + 0.4 μM of Aβ(1-42)*555) added to the culture medium. Then, the cells were washed once with 1 mL of MLocke’s K5, and the 35 mm dishes were placed in the holder of the microscope at 37 °C for RF imaging. The fluorescence microscopy images of the HT-22 cells were acquired with a Hamamatsu Orca-R^2^ CCD camera (binning mode 2 × 2) camera (Hamamatsu, Hamamatsu-city, Japan) attached to a Nikon Diaphot 300 epifluorescence microscope (Tokyo, Japan) with an NCF Plan ELWD 40× objective, using an excitation filter of 556 nm and a dichroic mirror of 580 nm with an emission filter of 590 nm, and a 0.06 s exposure time. The ROI tool of the HCImage software was used for the quantitative analysis. The average fluorescence intensity readings per pixel in HT-22 cells were taken from several fields for a total number of 80 cells and after the subtraction of the autofluorescence. In a parallel experiment, the average intensities per pixel obtained with the increasing concentrations of Aβ(1-42)*555 in fixed and permeabilized HT-22 cells were recorded for the calibration of the Aβ(1-42)*555 fluorescence under the same experimental conditions. 

### 4.7. FRET Imaging 

FRET imaging was performed as described in previous works of our laboratory [6,61,75]. First, the HT-22 cells were stained with different concentrations of Aβ(1-42)*555 (25–100 nM), to perform the measurements with the lowest Aβ(1-42)*555 concentrations, to highlight only the subcellular location of the high-affinity binding sites for Aβ(1-42). Fixed and permeabilized HT-22 cells were blocked with 1% bovine serum albumin in phosphate-buffered saline (PBS), supplemented with 0.2% 4-(1,1,3,3-tetramethyl butyl) phenyl-polyethylene glycol (Triton X-100^TM^) (PBST) for 1 h at room temperature (RT), and washed three times with PBS (the washing step). Then, the cells were incubated only with PBST for 2 h at 37 °C (to simulate the incubation with primary and secondary antibodies) and after the washing step, 25 nM of Aβ(1-42)*555 was added to the plate, and incubated for 15 at RT and with gentle mixing. The HT-22 cells were placed at the inverted epifluorescence microscope and the imaging acquisition was performed using an excitation filter of 556 nm and 470 nm, and a dichroic mirror of 580 nm with an emission filter of 590 nm (RF), and a 0.03 s exposure time. Afterwards, the same procedure was repeated for 50, 75, and 100 nM Aβ(1-42)*555. The following selected protein targets for the FRET imaging were used: PDI (goat anti-PDI, 1:50) and CaM (rabbit anti-CaM, 1:200). After blocking with 1% bovine serum albumin in PBST for 1 h, the HT-22 cells were incubated for 1 h with the respective primary antibodies (anti-PDI or anti-CaM) diluted in PBST. Next, the HT-22 cells were incubated for 1 h with the appropriate Alexa488-labeled secondary antibody in PBST (1:200) and washed again before the acquisition of the fluorescence microscopy images stained only with the donor dye. The image acquisition was performed with an excitation filter of 470 nm, and 510 nm dichroic mirror/520 nm emission filter (green fluorescence, GF). After finishing the acquisition of the FRET donor images, the HT-22 cells were incubated for 15 min at RT with 25, 50, or 100 nM Aβ(1-42)*555 in PBS with continuous and gentle mixing. The GF and RF imaging acquisitions were performed as described earlier. The contribution of HT-22 autofluorescence and a secondary Alexa488-antibody in the absence of the primary antibody were assessed before running the FRET experiments and were found to be lower than 5% of the average fluorescence intensity per pixel, obtained with specific primary antibodies. This background signal was subtracted for calculations of the RF/GF ratio obtained with the HT-22 plates.

### 4.8. Intracellular Cytosolic Calcium Measurements

Cytosolic Ca^2+^ imaging was assessed in the HT-22 cell culture with the fluorescent probe Fluo3-AM. Briefly, after cell seeding in 35 mm dishes, the HT-22 cells were incubated with 2 μM Aβ(1-42) for 2 h in a culture medium at 37 °C and 5% CO_2_, with continuous and gentle mixing. The untread cells were regarded as the controls. One hour before the end of the incubation with Aβ(1-42), the HT-22 cells were loaded with 5 μM of Fluo3-AM plus 0.025% Pluronic^®^ F-127 with continuous and gentle mixing. Then, the cells were washed once with 1 mL of MLocke’s K5 buffer, and the 35 mm culture dishes were placed in the thermostatic plate at 37 °C (Warner Instrument Co., Hamden, CT, USA) of the Nikon Diaphot 300 inverted epifluorescence microscope (Tokyo, Japan) with an NCF Plan ELWD 40× objective (the pixel size of the images shown in this work was 0.2 μm). The images of the Fluo3-loaded cells were acquired with the Hamamatsu Orca-R^2^ CCD camera (binning mode 2 × 2) with an excitation filter of 470 nm, a dichroic mirror of 510 nm with an emission filter of 520 nm, and a 0.3 s exposure time. The supplementary Figure S8 shows that the application of 100 μM of acetylcholine elicited intracellular calcium concentration peaks in the HT22 cells, but it is to be noted that this was observed in only less than 25% of the HT22 cells of the culture dish, and the majority of the cells displayed an almost negligible intracellular calcium response to acetylcholine or carbachol. The depletion of the Ca^2+^ stores was triggered by adding 2 μM of the SERCA pump blocker Tg plus ethylene glycol tetraacetic acid (1 mM), in a Ca^2+^-free Mlocke’s K5 buffer. Then, the SOCE was measured after the addition of 3 mM of CaCl_2_ to the Tg-containing medium. As a positive control of this experiment, we measured the SOCE in the presence of the selective inhibitor BTP2 (3 μM), preincubated for 15 min in the HT-22 culture media at 37 °C. The activity of the RyR Ca^2+^-leak channels was evaluated by incubation with 100 μM of the RyR antagonist ryanodine for 1 h in the HT-22 culture media. In all experiments with antagonists, we performed the respective dimethylsulfoxide vehicle controls. Additionally, the response of the SOCE was evaluated in the presence of 2 μM of scrambled Aβ(1-42) incubated for 2 h at 37 °C and 5% CO_2_.

The cytosolic Ca^2+^ imaging using Fura2-AM has been performed in Fura2-loaded cells, as described in our previous works [6,61,76].

### 4.9. Co-Immunoprecipitation 

The formation of the STIM1:Aβ(1-42) complexes was evaluated using the co-immunoprecipitation protocol, as described previously, but with some modifications [6]. The HT-22 cells were lysed in a buffer: 25 mM of Tris(hydroxymethyl)aminomethane–HCl, pH 7.4, 150 mM of NaCl, 5 mM of ethylenediaminetetraacetic acid, 50 mM of NaF, 5 mM of NaVO_3_, 0.25% of Triton X-100, and 5 mM of methyl-β-cyclodextrin, supplemented with 1× SIGMAFAST^TM^ protease inhibitor cocktail. After cell centrifugation at 2000× *g* for 2 min at 4 °C, the supernatant was collected and supplemented with 50% glycerol. Bradford’s method was used to measure the protein concentration in HT22 lysates using the bovine serum albumin as the standard. The co-immunoprecipitation was carried out using the protein A/G PLUS-Agarose, following the instructions described by the manufacturer in the technical data sheet. The co-immunoprecipitation experiments were performed with 500 μg of HT-22 lysate in the absence or presence of Ca^2+^ and with 1 μM of the peptide VFAFAMAFML(amidated-C-terminus amino acid), to fully antagonize the Aβ(1-42):CaM complexation [8], previously incubated for 30 min. Then, 10 μg of mouse anti-β-amyloid antibody was added to the Eppendorf tube and incubated for 1 h at 4 °C with continuous gentle shaking. Afterward, 50 μL of protein A/G PLUS-Agarose was added and incubated overnight at 4 °C with continuous shaking. The next day, the PBS control sample or 0.25 μM of Aβ(1-42) in the PBS (treated sample) was added and incubated for 1 h at 4 °C with continuous shaking. The agarose beads were precipitated by centrifugation at 2500× *g* for 5 min at 4 °C in a refrigerated Eppendorf microcentrifuge. Then, the precipitated beads were subjected to three washes with 50 μL of PBS (control sample) or 50 μL of PBS plus 0.25 μM of Aβ(1-42) (treated sample). A centrifugation step (2500× *g*, 5 min at 4 °C) was performed in a refrigerated Eppendorf microcentrifuge after each washing step. The supernatant was carefully removed, and the beads resuspended in 25 μL of electrophoresis sample buffer, boiled for 4 min and stored at −20 °C, until running on a sodium dodecyl sulfate-polyacrylamide gel electrophoresis (SDS-PAGE) for Western blotting analysis.

### 4.10. Western Blotting 

The SDS-PAGE was run at a concentration of 7.5% acrylamide, using 20 μL of co-immunoprecipitated sample per lane. The gels were transferred to polyvinylidene difluoride membranes of a 0.2 μm pore size and blocked with 3% bovine serum albumin in Tris(hydroxymethyl)aminomethane-buffered saline supplemented with 0.05% polyoxyethylene sorbitan monolaurate (Tween 20^TM^) (TBST) for 1 h at RT. The membranes were washed three times with TBST (washing step) and incubated with the primary antibody anti-STIM1 (1μg/mL) diluted in TBST for 1 h at RT. After the washing step, the membranes were incubated with the anti-sheep secondary IgG antibody conjugated with horseradish peroxidase at a dilution of 1:2000 in TBST for 1 h at RT. Then, the membranes were washed three times with TBST, followed by incubation for 3 min with the Bio-Rad Clarity Western ECL substrate. The Western blots were revealed with Bio-Rad ChemiDoc^TM^ XRS+ (Bio-Rad, Hercules, CA, USA) and data were analyzed with Image Lab 6.0.1 software.

### 4.11. STIM1-GFP Constructs and Expression 

The cDNA coding for the human STIM1 transcript variant 2 (NM_003156) was cloned as a *Bam*HI*-Not*I insert into pcDNA5-FRT/TO vector (Thermo Fisher Scientific) carrying a C-terminal green fluorescent protein (GFP) tag, as described previously [77]. The generation of Flp-InTM T-RExTM HEK293 cells, able to inducibly express tagged STIM1, was performed as described elsewhere [77]. The HEK293 cells were cultured on 10 cm diameter dishes in DMEM with 10% (*v*/*v*) fetal bovine serum, 2 mM of L-glutamine, 100 U/mL of penicillin, 0.1 mg/mL of streptomycin, 100 μg/mL of hygromycin B, and 15 μg/mL of blasticidin in a humidified atmosphere of air and CO_2_ at 37 °C. The cells were treated with 1 μg/mL doxycycline for 22–24 h to induce the expression of the tagged STIM1.

In addition, a HEK293 cell line was generated to inducibly express a variant of STIM1-GFP with a deletion of the amino acids 235–442 (STIM1-Δ235-442-GFP). The generation of the construct for the expression of this variant was performed by an overlap extension polymerase chain reaction (PCR) mutagenesis, using the vector described above as a template. All constructs were sequenced at the DNA sequencing facility of the Universidad de Extremadura. 

### 4.12. Fluorescence Measurements and Calculation of the FRET Parameters J and R_0_

The binding between STIM1 and Aβ(1-42) was measured using samples of the membrane fraction of the hypotonic lysates of the HEK293 cells expressing STIM1-GFP. The HEK293 cells were lysed as described in [78], but with small modifications. Briefly, the cells were washed with ice-cold PBS and swelled in a hypotonic buffer (10 mM HEPES pH 7.4). After 15 min, 5× *g* of buffer was added to achieve a final concentration of 50 mM HEPES pH 7.4, 150 mM of NaCl, 5 mM of MgCl_2_, 0.5 mM of dithiothreitol, 1 mM of benzamidine, 0.1 mM of phenylmethylsulfonyl fluoride, 2 mM of sodium orthovanadate, 5 mM of sodium fluoride, 5 mM of sodium pyrophosphate, and 10 mM of beta-glycerophosphate. The cell suspension was passed 20 times through a 25 g syringe. The nuclei were pelleted by centrifugation at 1000× *g* for 5 min at 4 °C. The non-nuclear supernatant was centrifuged at 100,000 g for 20 min, and the resulting supernatant was saved as the cytosol fraction. The membrane pellets were resuspended in 100 mM of KCl, 50 mM of HEPES, pH 7,4, and 50% glycerol was added to samples before storing at −80 °C.

The effect of the titration of STIM1-GFP-containing membranes with different concentrations of Aβ(1-42)*555 (10-30 nM) was measured in the presence of 50 μM of Ca^2+^ and a Ca^2+^-free medium. In addition, a STIM1-GFP construct of STIM1 with the deletion of the amino acids 235–442 of the cytosolic domain, where the CaM-binding domain of the STIM1 has been located [51], was also assessed and the GFP-empty construct was used as the control. 

The fluorescence measurements were performed using a Fluoromax+ fluorescence Spectrophotometer (Jovin Yvon technologies) at RT (24–25 °C) in quartz cells of 1 cm light-path length, with excitation and emission slits set to 2 nm. The FRET parameters J and R_0_ for the donor/acceptor pair STIM1-GFP/Aβ(1-42)*555 have been measured and calculated, as done for other FRET pairs in previous works of our laboratory [79,80]. Using a value of 0.6 for the quantum yield of GFP [81], we obtained an R_0_ value of 5.7 nm. However, it is to be noted that the anisotropy of the STIM1-GFP fluorescence emission was found to be relatively high, 0.656 ± 0.006, pointing out that there is relatively large uncertainty in this value of R_0_, which was calculated assuming random orientation between the donor and acceptor (see [82]). 

### 4.13. Statistical Analysis 

Statistical analysis was carried out by Student’s *t*-test and the results were expressed as the mean ± SEM. A significant difference was accepted at the *p* < 0.05 level. All results were done at least in 8–10 Petri plates in 4–5 independent assays (n > 60–80 cells) for each condition.

## Data Availability

Not applicable.

## Supplementary Materials

The following supporting information can be downloaded at: https://www.mdpi.com/article/10.3390/ijms232012678/s1.

## Abbreviations

Aβ	amyloid β peptide
Aβ(1-42)*555	Aβ(1-42)-HiLyte™-Fluor555
AD	Alzheimer’s disease
Anti-CaM*A488	complex anti-CaM + AlexaFluor488-labeled anti-IgG antibody
Anti-PDI*A488	complex anti-PDI + AlexaFluor488-labeled anti-IgG antibody
a.u.	fluorescence intensity in arbitrary units
BTP2	N-[4-[3,5-bis(trifluoromethyl)pyrazol-1-yl]phenyl]-4-methylthiadiazole-5-carboxamide
CaM	calmodulin
CaMBD	calmodulin binding domain
DMEM	Dulbecco’s modified Eagle’s medium
ER	endoplasmic reticulum
FCCP	trifluoromethoxy carbonylcyanide phenylhydrazone
Fluo3-AM	Fluo-3-pentaacetoxymethyl ester
FRET	fluorescence resonance energy transfer
Fura2-AM	Fura2 acetoxymethyl ester
GF	green fluorescence
GFP	green fluorescent protein
GSH	reduced glutathione
H_2_DCFDA	2′,7′-dichlorodihydrofluorescein diacetate
HEPES	N-[2-hydroxyethyl] piperazine-N′-[2-ethanesulfonic acid]
IC50	concentration producing 50% of the maximum effect
IgG	immunoglobulin G
IP3R	inositol 1,4,5-trisphosphate receptor
MCB	monocholorobimane
MTT	3-(4,5-dimethylthiazol-2-yl)-2,5-diphenyltetrazolium bromide
PBS	phosphate-buffered saline
PBST	PBS supplemented with 0.2% 4-(1,1,3,3-tetramethyl butyl) phenyl-polyethyleneglycol
PDI	protein disulfide isomerase
RF	red fluorescence
RT	room temperature
ROS	reactive oxygen species
RyR	ryanodine receptor
SDS-PAGE	sodium dodecyl sulfate-polyacrylamide gel electrophoresis
SEM	standard error of the mean
SERCA	Sarco-endoplasmic reticulum Ca^2+^-ATPase
SOCE	store-operated calcium entry
STIM1	stromal interaction molecule 1
TBST	Tris(hydroxymethyl)aminomethane-buffered saline supplemented with 0.05% polyoxyethylene sorbitan monolaurate
Tg	thapsigargin
TMRE	Tetramethylrhodamine ethyl ester
XeC	Xestonpongin C
