# Supplementary material for: Internalized Amyloid-β (1-42) Peptide Inhibits the Store-Operated Calcium Entry in HT-22 Cells
Source: Int J Mol Sci. 2022 Oct 21;23(20):12678. doi: 10.3390/ijms232012678 (PMC9604325; doi:10.3390/ijms232012678)
Supplement: Supplementary file 1 [file ijms-23-12678-s001.zip › ijms-1941218-supplementary.pdf]

Supplementary Figure S1. IJMS-1941218

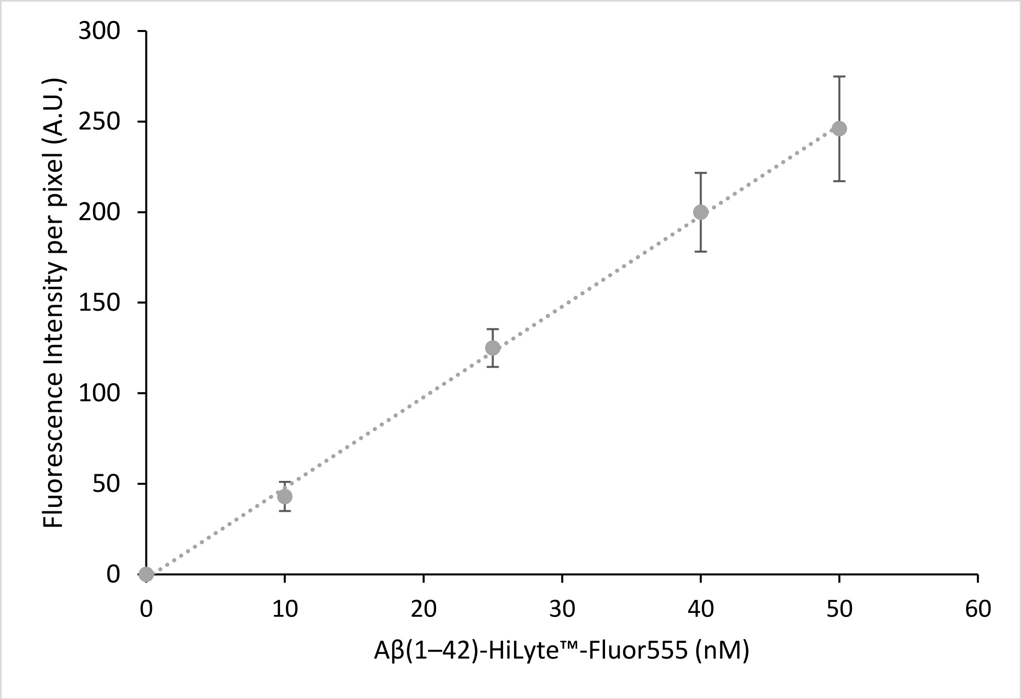

Supplementary Figure S1. Calibration data were obtained with different Aβ(1-42)*555 concentrations added to the extracellular medium of fixed and permeabilized HT-22 cells to calculate the internalized concentration of Aβ(1–42) in living HT-22 cells. The concentration of Aβ(1-42)*555 is expressed as nanomoles of monomers/L. The results shown are the mean ± SEM of triplicate experiments.

Supplementary Figure S2. IJMS-1941218

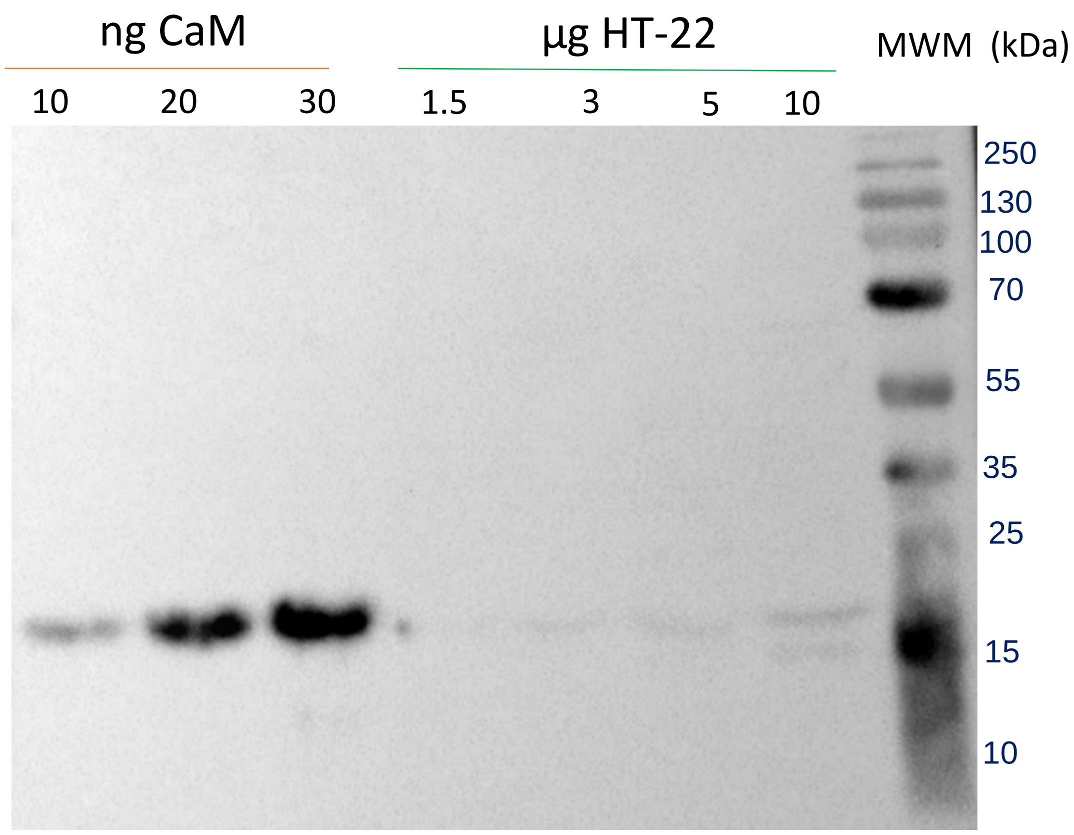

Supplementary Figure S2. Quantification of the content of CaM in HT-22 cells by Western blotting. CaM has been quantified in polyvinylidene difluoride membranes using an anti-CaM antibody (Epitomics 1716-1). Calibration has been performed using human recombinant CaM at the amounts indicated at the top of the CaM lanes, 10, 20, and 30 ng. The micrograms (µg) of protein of HT-22 cell lysates are indicated at the top of the corresponding lanes. The molecular weight of the protein markers lane (MWM) is included on the right-hand side of the Western blot. The intensity of the CaM bands has been calculated with the Image JTM software, using proximal areas of the same size for background subtraction.

Supplementary Figure S3. IJMS-1941218

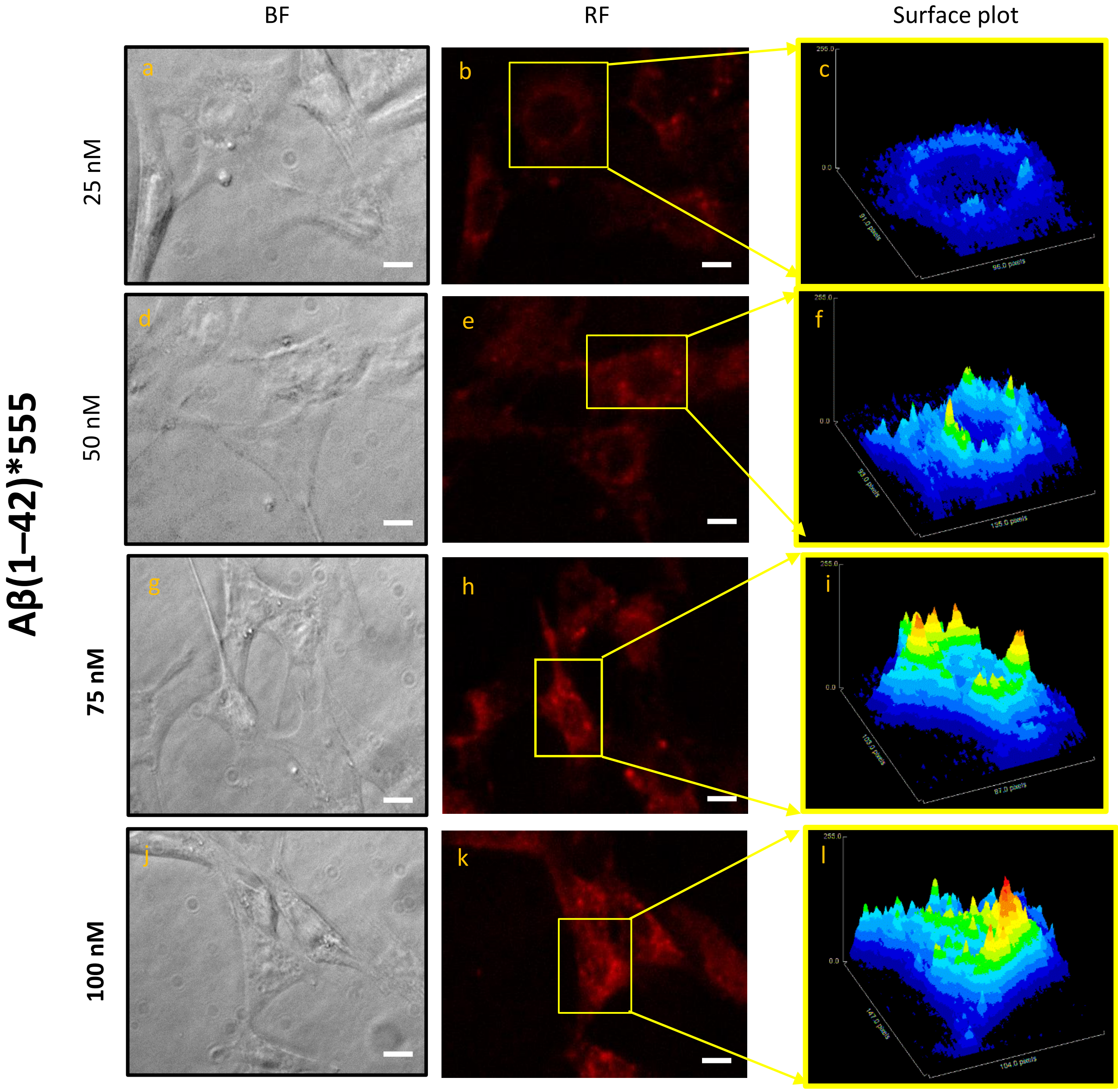

Supplementary Figure S3. Fluorescence microscopy images of fixed HT-22 stained with increased concentrations of Aβ(1–42)*555. Representative fluorescence microscopy images of permeabilized HT-22 staining with 25, 50, 75, and 100 nM of Aβ(1–42)*555. BF and RF images are shown for representative selected fields. Surface plots are shown for each concentration tested, highlighting the focalized subcellular distribution of Aβ(1–42) mainly in the perinuclear region of HT-22 soma. The exposure time for RF images was 0.03 s. Scale bar inserted in fluorescence microscopy images = 20 μm

Supplementary Figure S4. IJMS-1941218

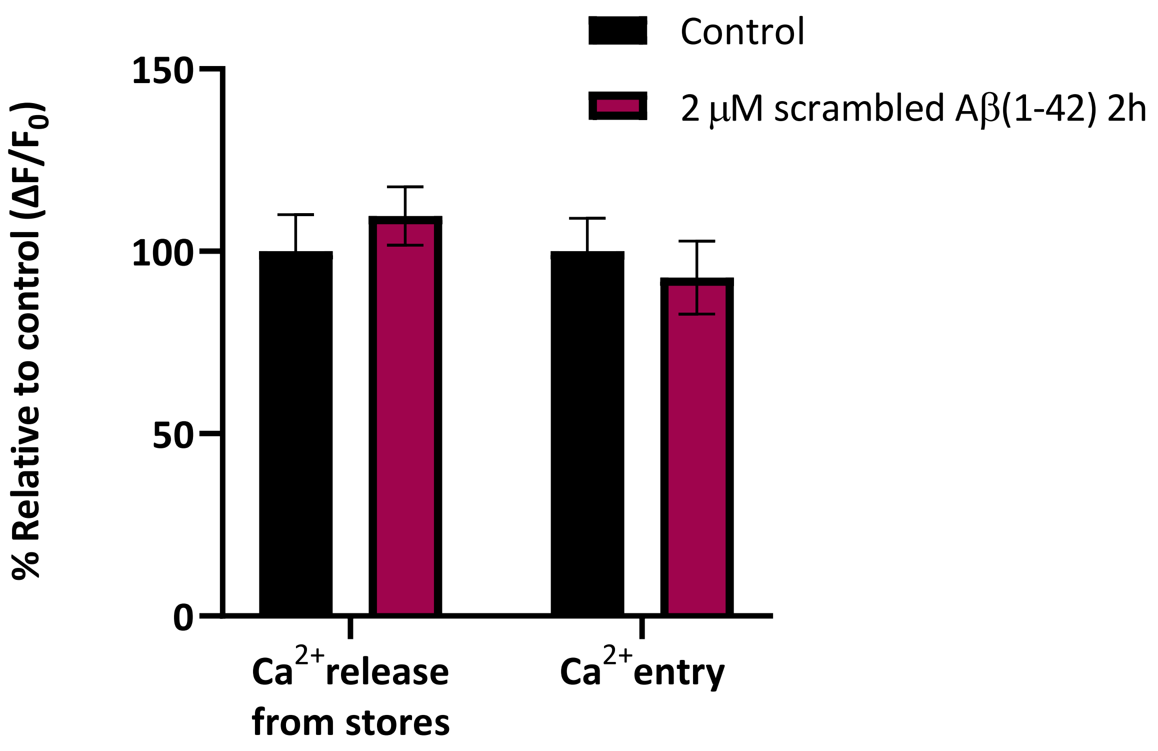

Supplementary Figure S4. Scrambled Aβ(1-42) did not alter the resting cytosolic Ca^{2+} concentration peaks in the SOCE experiment. Untreated cells (control) and HT-22 treated with 2 µM scrambled Aβ(1-42) for 2 h at 37 °C and 5% CO_2 were loaded with Fluo3-AM plus Pluronic® F-127 and subjected to SOCE experiments as described in the Materials and Methods section. The results presented do not show statistically significant differences in Ca^{2+} release from stores after the addition of Tg or in the Ca^{2+} influx after Ca^{2+} addition to the extracellular medium. Data are presented as the means ±SEM of the fluorescent intensity (ΔF/F0) represented by percentage (%), relative to control cells, of experiments done at least in 8 Petri plates in 4 independent assays (n>60 cells). n.s. – non significant ($p > 0.05$).

Supplementary Figure S5. IJMS-1941218

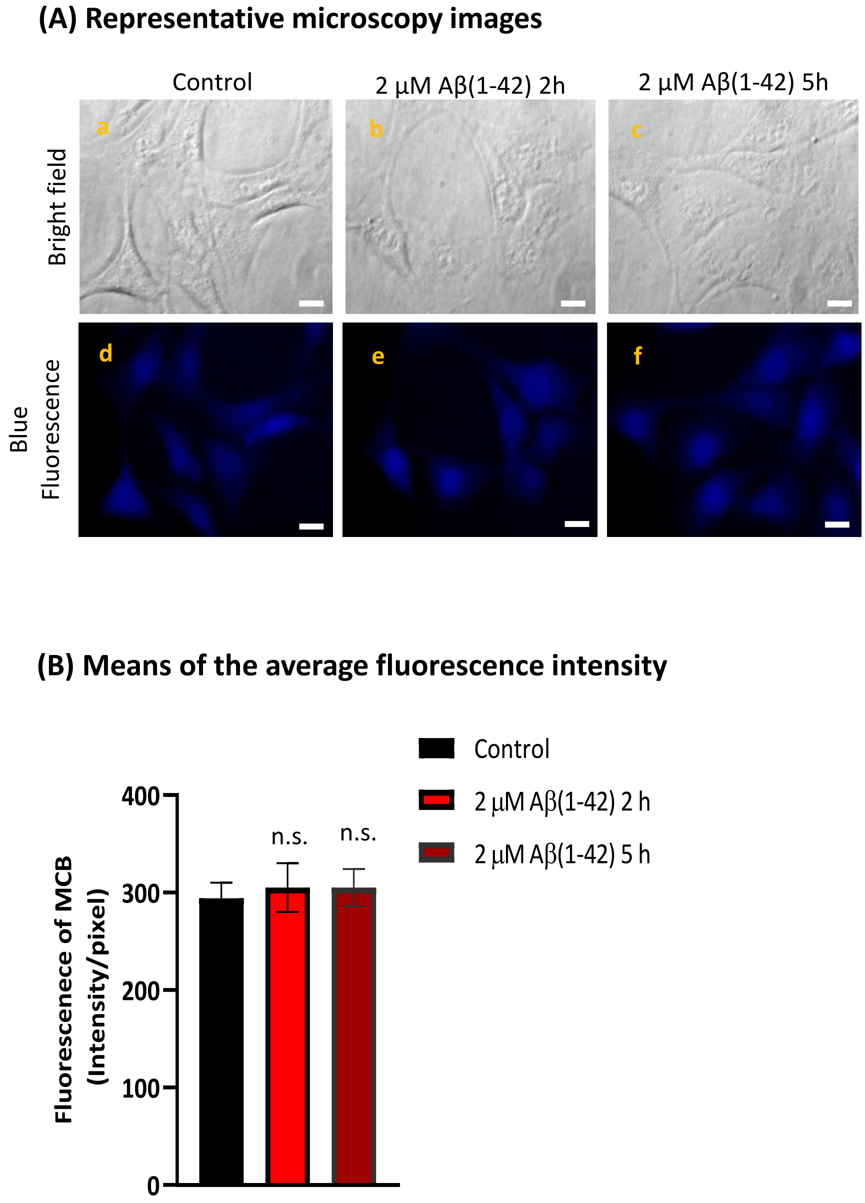

Supplementary Figure S5. GSH levels are not altered after 2 h and 5 h incubation with 2 μM Aβ(1-42) in HT-22 cells. Untreated HT-22 cells and cells treated with 2 μM Aβ(1-42) up to 5 h at 37 ºC were washed once with MLocke's K5 buffer and the plates were placed in the holder of the fluorescence microscope (37 ºC) for cell imaging before and after the addition of 10 μM MCB as described in the Material and Methods section.

Panel A: representative bright field and blue fluorescence images (GS-MCB) acquired 2 min after MCB addition of untreated cells HT-22 (control) and treated cells with 2 μM Aβ(1-42). The time of exposure was 0.7 s and the scale bar inserted = 20 μm. Panel B: means of fluorescence intensity per pixel of HT-22 cells after 2 min of incubation with MCB (10 μM). The results shown are the mean ± SEM. (*) $p<0.05$. The results show no statistical difference in GSH levels between untreated cells (control) and cells treated with 2 μM Aβ(1-42) for 2 h and 5 h at 37 ºC (n.s.- non-significant).

Supplementary Figure S6. IJMS-1941218

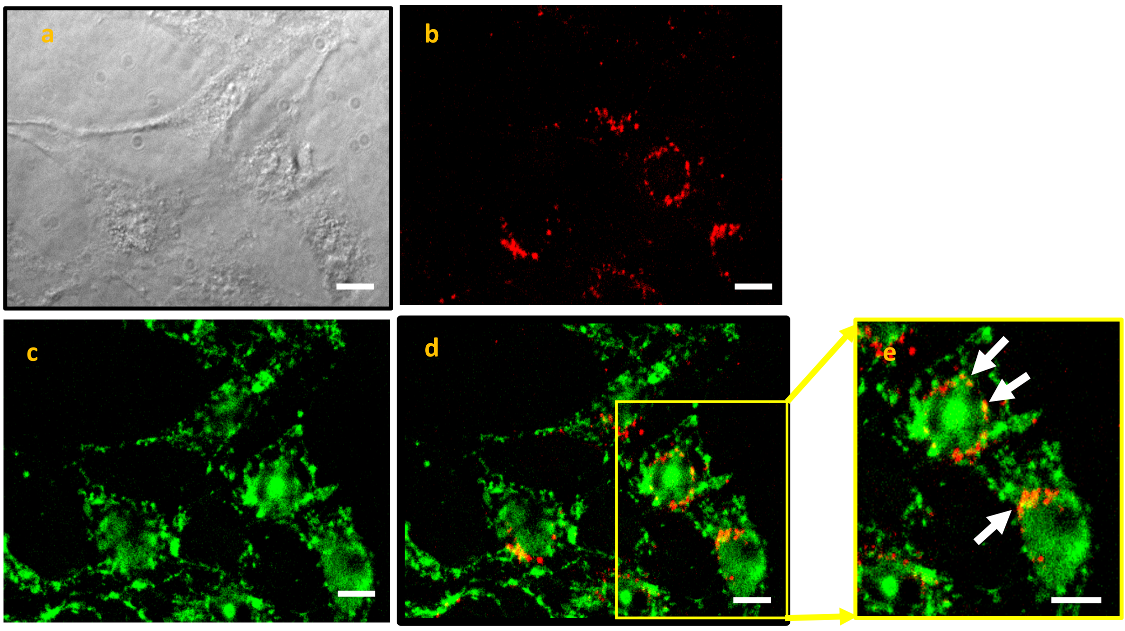

Supplementary Figure S6. Marginal co-localization between internalized Aβ(1–42)*555 and mitochondria, after 5 h of Aβ(1–42) incubation in HT-22 cell line. HT-22 cells were incubated with 2 μM Aβ(1–42) (1.8 μM of Aβ(1–42) plus 0.2 μM of Aβ(1–42)*555) for 5 h at 37 ºC and 5% CO_2. Next, cells were washed with MLocke's K5 buffer and placed in the holder of the fluorescence microscope thermostated at 37 ºC for fluorescence imaging of Aβ(1–42)*555 (b). Then, cells were incubated with 20 nM MitoTracker™ Green FM for 5 min at 37 ºC and 5% CO_2 and placed again in the holder of the fluorescence microscope for fluorescence imaging acquisition (c). Figure (d) shows a merged image between Aβ(1–42)*555 and MitoTracker™ Green FM. Figure (e) shows a focalized zoom of Figure (d) and the white arrows point out regions of higher co-localization between Aβ(1–42)*555 and MitoTracker™ Green FM. Figure (a) shows the bright field of the selected representative image. Scale bar inserted in fluorescence microscopy images = 20 μm.

Supplementary Figure S7. IJMS-1941218

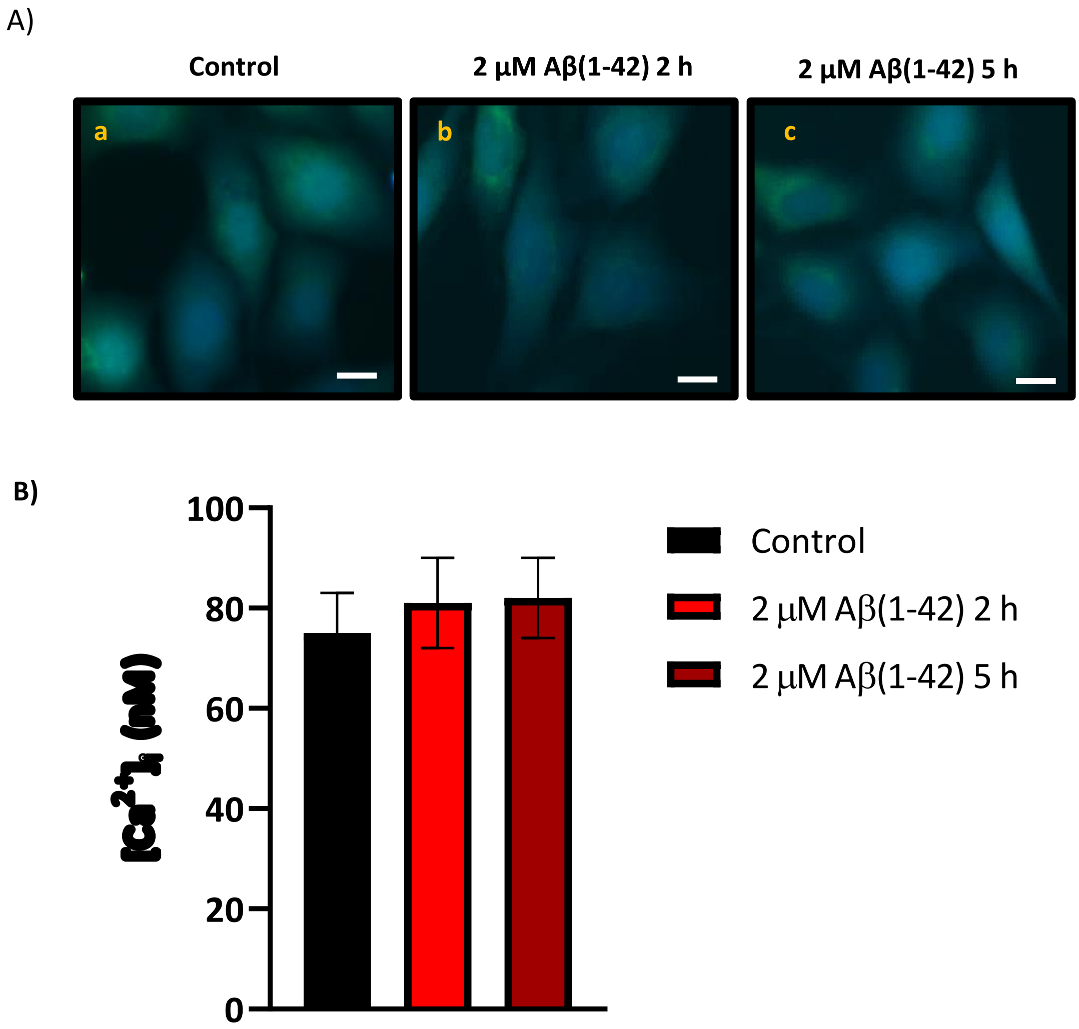

Supplementary Figure S7. Intracellular Ca^{2+} concentration ($[Ca^{2+}]i$) is not significantly altered in HT-22 cells after 2 h and 5 h incubation with 2 μM Aβ(1-42). Untreated (control) and treated HT-22 cells with 2 μM Aβ(1-42) for 2 h and 5 h at 37 ^{o}C were loaded with 5 μM Fura2-AM or Fluo3-AM plus 0.025 Pluronic® F-127 for [Ca2+]i measurements.
A) Representative fluorescence images of Fura2-loaded untreated HT-22 cells (control, a) and HT-22 cells treated with 2 μM Aβ(1-42) for 2 h (b) and 5 h (c). Scale bar = 20μm.
B) $[Ca^{2+}]i$ obtained for untreated cells (control, 75 $\pm$ 8nM) and HT-22 cells incubated with 2 μM Aβ(1-42) for 2 h (81 $\pm$ 9nM) and 5 h (82 $\pm$ 8nM). These results are the means $\pm$SEM of triplicate experiments (n>60 cells for each condition).

Supplementary Figure S8. IJMS-1941218

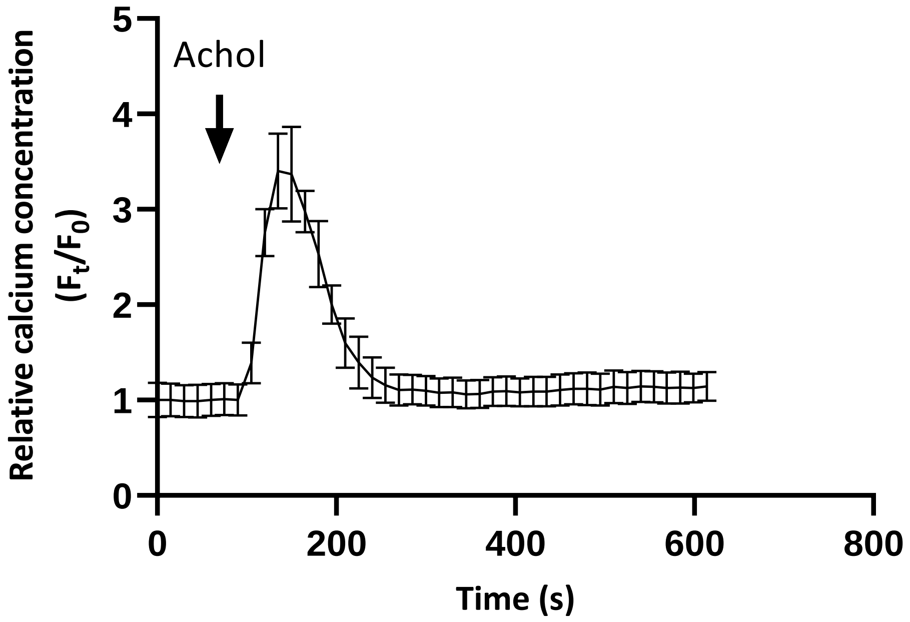

Supplementary Figure S8. Representative kinetic traces of the average fluorescence intensity of Fluo3-loaded HT22 cells (Ft/F_0) recorded over time every 15 s intervals after the addition of 100 μM acetylcholine (Achol) shows Ca^{2+} entry mediated by cholinergic receptors. The data shown are the means ±SEM of n =16 cells.
